# Small Area Variations in Dietary Diversity Among Children in India: A Multilevel Analysis of 6–23-Month-Old Children

**DOI:** 10.3389/fnut.2021.791509

**Published:** 2022-02-16

**Authors:** Anoop Jain, Weiyu Wang, K. S. James, Rakesh Sarwal, Rockli Kim, S. V. Subramanian

**Affiliations:** ^1^Global Health and Social Medicine, Harvard Medical School, Boston, MA, United States; ^2^Harvard Center for Population and Development Studies, Cambridge, MA, United States; ^3^International Institute for Population Sciences, Mumbai, India; ^4^National Institution for Transforming India (NITI) Aayog, Government of India, New Delhi, India; ^5^Division of Health Policy and Management, College of Health Science, Korea University, Seoul, South Korea; ^6^Department of Social and Behavioral Sciences, Harvard T.H. Chan School of Public Health, Boston, MA, United States

**Keywords:** India, undernutrition, dietary intake, dietary diversity, multilevel modeling

## Abstract

Dietary diversity is an important indicator of child malnutrition. However, little is known about the geographic variation of diet indicators across India, particularly within districts and across states. As such, the purpose of this paper was to elucidate the small area variations in diet indicators between clusters within districts of India. Overall, we found that clusters were the largest source of variation for children not eating grains, roots, and tubers, legumes and nuts, dairy, vitamin A-rich vegetables and fruits, and other vegetables and fruits. We also found positive correlations between the district percent and cluster standard deviations of children not breastfeeding or eating grains, roots, and tubers, but negative correlations between the district percent and cluster standard deviation for the remaining seven outcomes. These findings underscore the importance of targeting clusters to improve child dietary diversity.

## Introduction

Dietary diversity is an important indicator of food consumption, and highlights the extent to which households have access to different food groups. Understanding dietary diversity is particularly important in the context of child health. Inadequate diets are an impediment to child survival, growth, and development (1). More specifically, the World Health Organization's indicator of minimum dietary diversity (MDD) for children between the ages of 6–23 months elucidates the micronutrient density of a child's diet. Additionally, examining diet indicators points to the specific food groups that might be missing from child diets. For example, one recent study shows that children who did not meet the MDD in India were less likely to consume flesh foods and vitamin A-rich vegetables and fruits (2). These results point to the specific types of foods that are missing from child diets, and can help inform intervention and policy design.

Yet, child undernutrition is typically quantified by anthropometric measures, such as height-for-age, weight-for-age, or weight-for-height (3, 4). This is despite the fact that one of the primary causes of child undernutrition is inadequate diet (5). While anthropometric measures do capture an individual child's genetics, environment, behavioral factors, and disease exposure (6), they do not always capture dietary deficiencies. Measuring MDD can highlight nutritional deficiencies among those children who do not meet the MDD, even if they do not experience a given form of anthropometric failure. In India for example, 78% of children between 6 and 23 months do not meet the MDD while 36% of children are stunted (7). Furthermore, vitamin-A deficiency among children under five is 30% higher than the prevalence of stunting (8). This exemplifies how anthropometry is not related to all forms of malnutrition, and that anthropometry does not always paint a clear picture of a child's nutritional deficiencies. Yet the inclusion of diet indicators as intrinsically important outcomes remains rare in research and policy in the context of child undernutrition (9–11).

The Indian government's National Nutrition Strategy (NNS) was created as a response to the fact that child malnutrition remains a major public health problem throughout India. The NNS aims to improve nutrition outcomes by 2022 by targeting districts with a high prevalence of undernutrition (12). One issue with the NNS, however, is its explicit focus on districts. In the context of child malnutrition, some of the geographic variation is attributable to between-district differences (13), while other studies have shown that a larger share of the variation is attributable to states (14, 15) and villages (16–18), a consequence of the geographic clustering of risk factors (19). Recent studies have also elucidated the small area variation of anthropometric failure within districts. For example, strong positive correlations between the mean and standard deviations of prevalence of stunting, underweight, and wasting have been found at the district level (20). This implies that districts with a high burden of child malnutrition also have considerable variation within the district, suggesting that in some cases, within district geographic units should be targeted instead of just the district at large. Within district inequality in India can further be exemplified by the fact that the largest share of poverty and child sex ratio is attributable to between-village variations (21, 22). These studies show the importance of states and within-district units in the context of anthropometric failure.

Given the World Health Organization's emphasis on starting complementary feeding at six months, an emerging body of literature has also started examining the geographic variation of diet indicators, such as MDD, throughout India. The MDD is a binary indicator used to measure the micronutrient density of children between the ages of 6–23 months (23). Children must have consumed five or more of eight food groups in the previous day to qualify as having met the MDD (24). The MDD is a useful tool as it can identify high-need populations while also predicting anthropometric failure (25–29). Initial efforts have been made to quantify the extent to which dietary diversity varies between regions in India, and possible explanations for these variations (30–32). Regional variations in female literacy, household socioeconomic status, religion and caste, and agricultural productivity explain why dietary diversity varies between states and districts (10, 33, 34). However, more research is required to understand small area varitions in diet indicators within districts and between villages given that these are the geographic units at which other markers of child malnutrition vary.

Thus, the purpose of this paper was to elucidate small area variations of MDD and each of the composite food groups (breastmilk, grains, legumes, dairy, flesh foods, eggs, vegetables, and other vegetables/fruits) in India. We used the fourth round of the National Family Health Survey (NHFS-4) from 2015 to 2016 to conduct this analysis. This research is novel and significant because while prior research has examined small area variations in measures of anthropometric failure in India, small area variations in diet indicators have yet to be quantified. Doing so could help better inform policy design and implementation by demonstrating within district heterogeneity in children's dietary diversity.

## Methods

### Data Source and Sample

We used NFHS-4 for this analysis. Households were selected using a stratified two-stage cluster sampling strategy. Clusters were defined as groups of adjacent households, and were the primary sampling units (PSUs). Rural and urban clusters were selected in the first stage of sampling. Clusters with more than 300 households were divided in to smaller groups of 100 to 150 households. Therefore, clusters were either an entire PSU, or a part of a PSU, from which the final households were selected as part of the second stage of sampling. A maximum of 22 household were selected from any given PSU in final sample.

Overall, the survey contained data from a total of 601,509 households from 28,522 rural and urban clusters, in all 640 districts, and all 36 states/union territories. As such, 699,686 women between the ages of 15–49 and 259,627 children under five were surveyed. However, the Demographic and Health Survey guidelines for calculating MDD are for mothers living with their youngest child between the ages of 6 and 23 months. As such, our final sample included 72,895 mothers currently living with their youngest child who was between 6 and 23 months old.

### Primary Outcomes

We analyzed the small area variation of a child not meeting the minimum dietary diversity (MDD) throughout all 36 states/union territories, 640 districts, and 25,121 urban/rural clusters. The MDD is constructed from a score between zero and eight assigned to each child between 6 and 23 months (24). One point is given for consuming one or more from eight food groups in the past 24 h. These eight food groups are: (a) breast milk (child is currently breastfeeding); (b) grains, roots, and tubers, which are comprised of bread, noodles, and other grains, or fortified infant food, or potatoes, cassava, or other tubers; (c) legumes and nuts, which are comprised of beans, peas, lentils, or nuts; (d) dairy, which is comprised of infant formula or tinned, powdered, or fresh milk, or cheese, yogurt, or other milk products; (e) flesh foods, which are comprised of liver, heart, or other organ meat, or fish/shellfish, or chicken, duck, or other birds, or any other meat; (f) eggs; (g) vitamin A-rich fruits and vegetables, which are comprised of pumpkins, carrots, or squash, or dark green leafy vegetables, or mangoes, papaya, and any other vitamin A-rich fruits; (h) any other fruits and vegetables. Children with a score of five or higher were classified as meeting the MDD, while those with scores below five were classified as not meeting the MDD (24). Until 2017, meeting the MDD was defined as eating four of seven groups. However, a panel of technical experts from the World Health Organization and UNICEF decided to include breast milk as a food group for 6–23 month old children. As such, meeting the MDD shifted from eating four of seven food groups to eating five of eight food groups (24). This is important considering that in their updated guidance, the World Health Organization and UNICEF have started to emphasize the timely commencement of complementary feeding after a child is 6 months old (24).

In addition to analyzing the small area variation of not meeting the MDD, we analyzed the small area variation for each of the component food groups that makes up the MDD. As such, we had a total of nine outcomes: (a) not meeting the MDD; (b) not currently breastfeeding; (c) did not eat grains, roots, and tubers; (d) did not eat legumes and nuts; (e) did not consume dairy; (f) did not eat flesh foods; (g) did not eat eggs; (h) did not eat vitamin A-rich fruits and vegetables; (i) did not eat other fruits and vegetables. Thus, we partitioned geographic variation in each outcome and elucidated the foods most needed by children in any given place (2).

### Statistical Analysis

The NFHS-4 data are structured such that children at level one were nested in clusters at level two, districts at level three, and states at level four. As such, we estimated a total of nine four-level variance component models to first decompose the total geographic variation for clusters, districts, and states for the probability of a child *i* in cluster *j*, district *k*, and state *l* not meeting the MDD or not eating each of the eight component food groups using equation (1) $logit\left({\mathrm{Pr}}_{ijkl}\right)=\;\beta_{0\;}+\left(u_{0jkl}+v_{0kl}+f_{0l}\right)$. In this model, β_0_represents the constant, while the random effects are the residual differentials for clusters *j* (*u*_0*jkl*_), districts *k* (*v*_0*kl*_), and states *l* (*f*_0*l*_). Each of the residual differentials is assumed to be normally distributed (see Supplementary Figures 1–9), with a mean of zero and a variance of *u*_0*jkl*_ ~ N(0,$\sigma_{u0}^{2}$), *v*_0*kl*_ ~ N(0, $\sigma_{v0}^{2}$), and *f*_0*l*_ ~ N(0, $\sigma_{f0}^{2}$) where the variances quantify the between-cluster (*u*_0*jkl*_), between-district (*v*_0*kl*_), and between-state (*f*_0*l*_) variation. The variance at level one (children) is assumed to be a constant in binary models (21, 35). We conducted this analysis in MLwiN 3.05 using the Monte Carlo Markov Chains method with a burn-in of 500 cycles and monitoring of 5,000 iterations of chains.

We then calculated the proportion of geographic variation attributable to clusters, districts, and states for each of the nine outcomes by dividing the variance of a given level by the total geographic variation (i.e., for the cluster level, $\sigma_{u0}^{2}$/($\sigma_{u0}^{2}$ + $\sigma_{v0}^{2}$ + $\sigma_{f0}^{2}$) * 100).

Next, we generated precision-weighted estimates specific to each cluster for each outcome. The percentage of each child in the cluster not meeting the MDD or eating the specific food group was calculated using equation (2) (exp[β_0_ + (*u*_0*jkl*_ + *v*_0*kl*_ + *f*_0*l*_)]/[1 + exp(β_0_ + (*u*_0*jkl*_ + *v*_0*kl*_ + *f*_0*l*_)]) * 100. We calculated the standard deviations of these cluster percentages by district, which would be used to elucidate the small area variation for each outcome. We also generated precision-weighted estimates specific to each district for each outcome. The percentage of each child in the district not meeting the MDD or eating the specific food group was calculated using equation (3) (exp[β_0_ + (*v*_0*kl*_ + *f*_0*l*_)]/[1 + exp(β_0_ + (*v*_0*kl*_ + *f*_0*l*_)]) * 100.

## Results

### Sample Characteristics

Our sample retained data from 25,121 out of the 28,522 clusters included in the full NFHS-4 dataset. On average, there were four children between the ages of 6–23 months per cluster, while the minimum was one and the maximum was 13. Of the 72,895 children in our sample from these clusters, 14,402 (19.8%) met the MDD, while 58,493 (80.2%) did not. Additionally, of the 72,895 children in our sample, a total of 10,603 children were not breastfed in the past 24-h and 22,364 children did not eat grains, roots, or tubers. Overall, 62,513 children from the whole sample did not eat legumes and nuts, 37,403 did not eat dairy, 64,403 did not eat flesh foods, and 62,323 did not eat eggs in the past day. Finally, of the total 72,895 children, 43,125 did not eat vitamin A-rich fruits and vegetables, while 55,424 did not eat other fruits and vegetables in the previous day.

### Relative Importance of Geographic Levels

**Figure 1:**
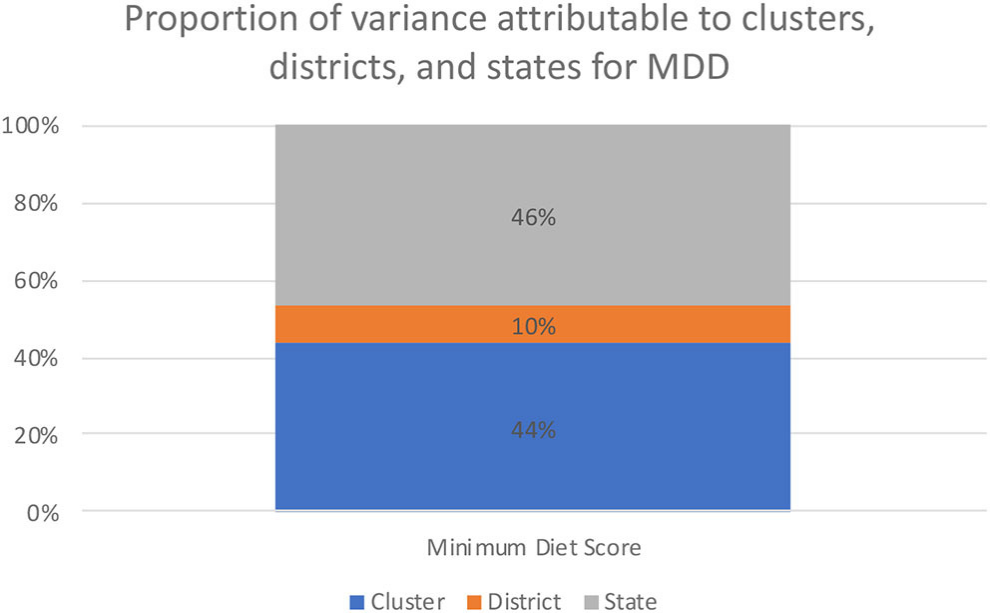
Variance partitioned between clusters, districts, and states for not meeting the minimum dietary diversity.

**Figure 2:**
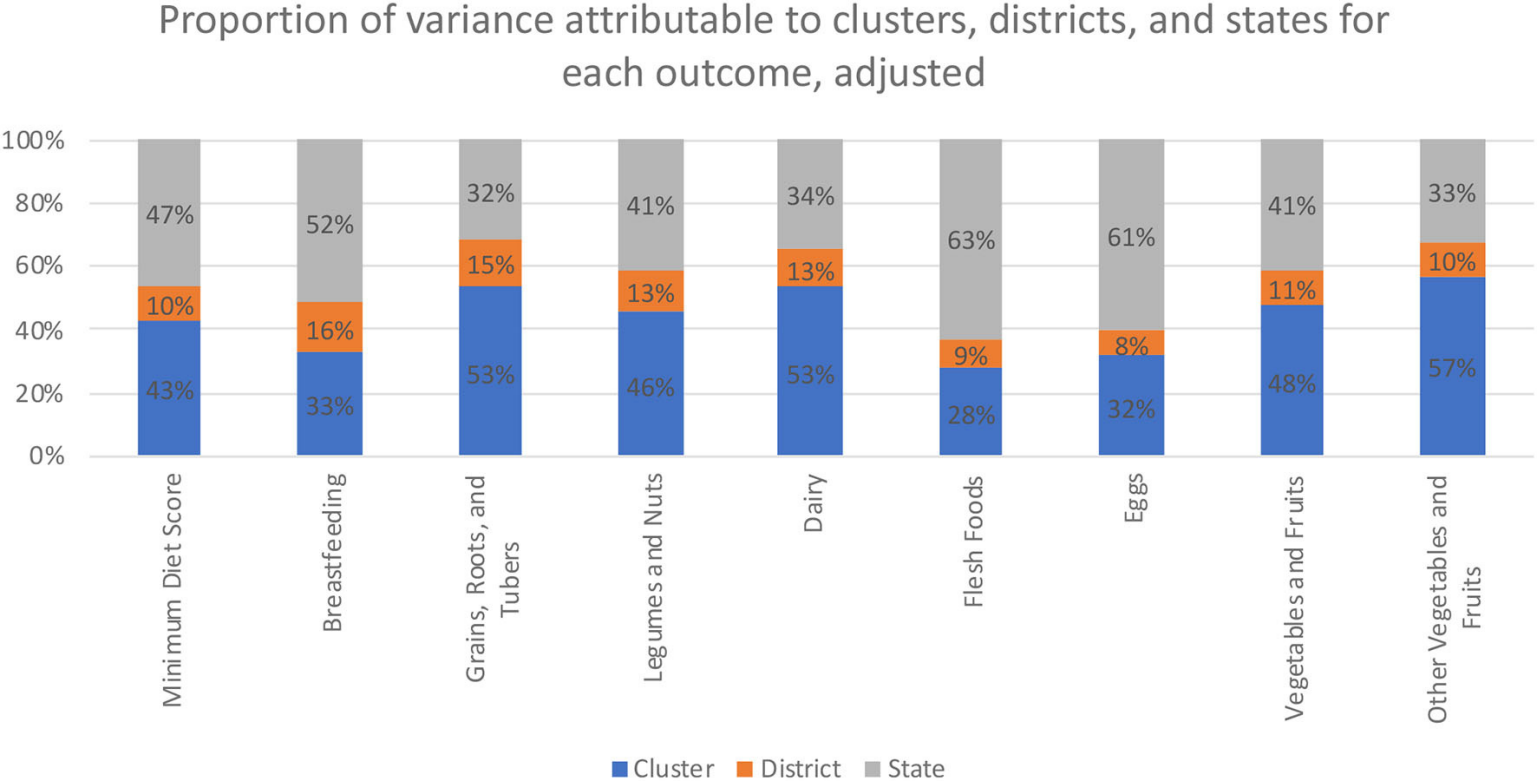
Adjusted variance partitioned between clusters, districts, and states for not consuming each food group.

Overall, we found that clusters were the largest source of geographic variation for the following outcomes: (a) grains, roots, and tubers (55%); (b) legumes and nuts (47%); (c) dairy (49%); (d) vitamin A-rich vegetables and fruits (49%); (e) other vegetables and fruits (55%). States were the largest source of geographic variation for the following outcomes: (a) total MDD (46%); (b) breastfeeding (51%); (c) flesh foods (65%); (d) eggs (62%). Districts accounted for the lowest source of geographic variation for all nine outcomes. These values are presented in Figure 1 and Supplementary Figure 10. After examining the percent of geographic variation explained by certain covariates associated with child malnutrition (26, 34), clusters were the largest source of geographic variation for these same outcomes even after adjusting for child's sex, household wealth, household caste, household religion, mother's education, and household location (urban/rural). These results are presented in Table 1 and Figure 2.

**Table 1:** Percent of cluster variation explained by adjusted model.

	**Minimum diet score**	**Breastfeeding**	**Grains, roots, and tubers**	**Legumes and nuts**	**Dairy**	**Flesh foods**	**Eggs**	**Vegetables and fruits**	**Other vegetables and fruits**
**Cluster (unadjusted)**	0.79	0.27	0.41	1.02	0.51	0.86	0.92	0.46	0.63
**Cluster (adjusted)**	0.77	0.24	0.39	1.04	0.45	0.87	0.94	0.47	0.61
**Percent change**	3%	11%	5%	−2%	12%	−1%	−2%	−2%	3%

### Small Area Variation in Child Dietary Diversity

**Figure 3:**
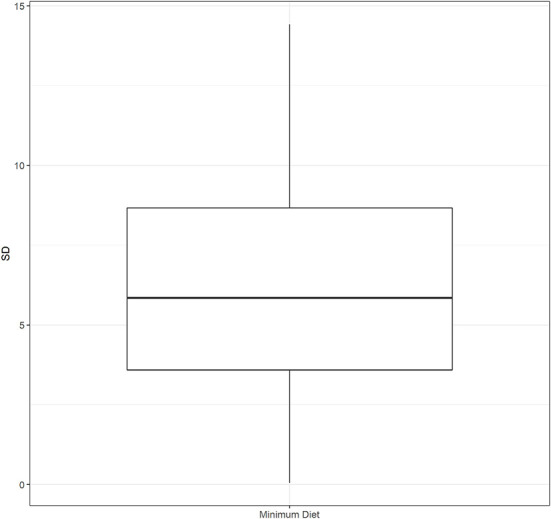
Distribution of standard deviations for not meeting the minimum dietary diversity.

**Figure 4:**
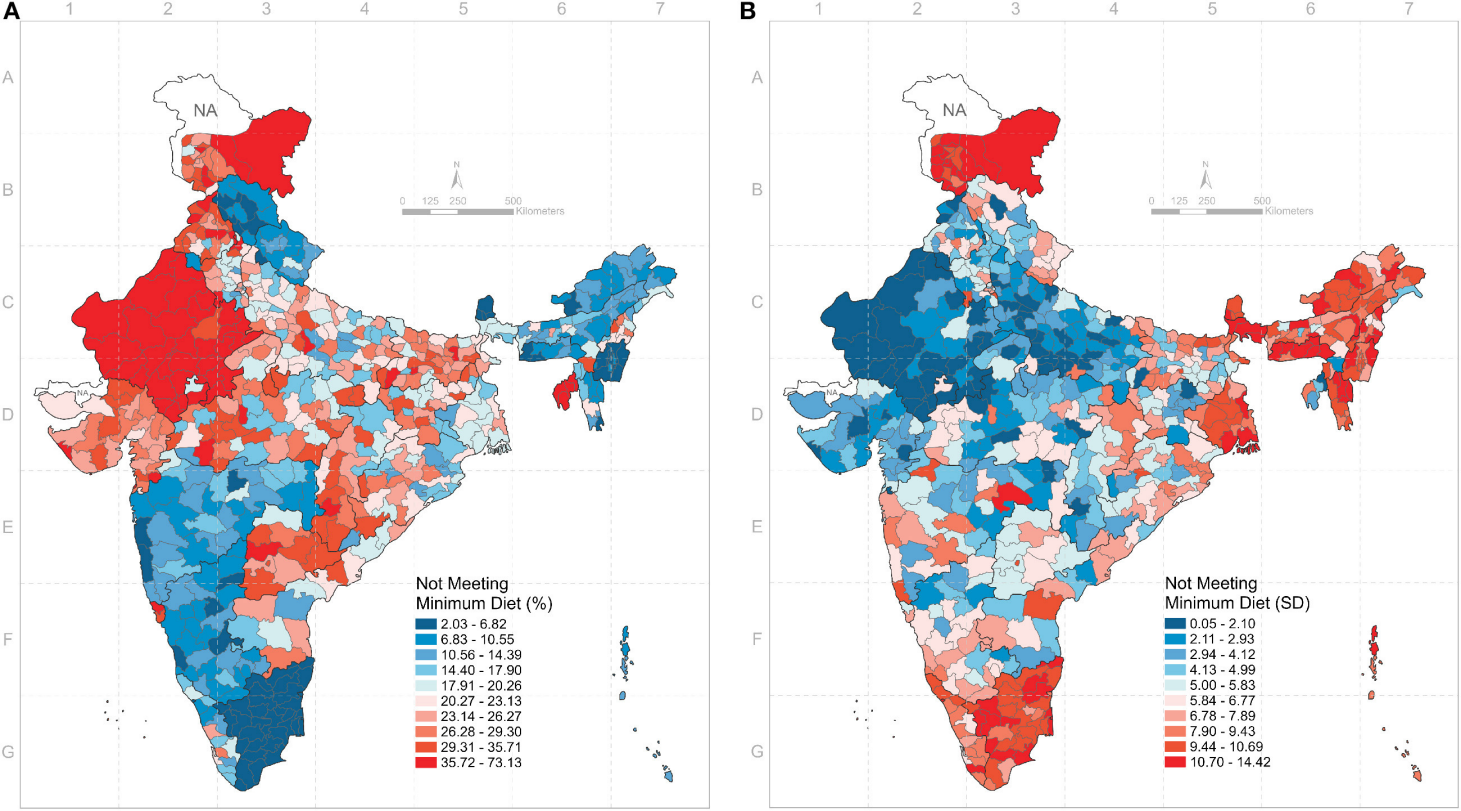
**(A)** Geographic distribution of percent children not meeting the minimum dietary diversity across 640 districts in India. **(B)** Geographic distribution of within-district, between-cluster standard deviation in percent children not meeting the minimum dietary diversity across 640 districts in India.

We computed the cluster standard deviations (SD) of the percentages of each outcome by district. These values quantified the within-district variation, or the small area variation, for each outcome. For the overall MDD, the SD ranged from 0.05 to 14.4 (median 5.7). For breastfeeding, the SD ranged from 0.08 to 5.3 (median 1.7). The SD for grains, roots, and tubers ranged from 1.4 to 9.6 (median 5.4), and the SD for legumes and nuts ranged from 0.02 to 16.7 (median 4.9). For dairy, the SD ranged from 2.8 to 12.5 (median 7.6) and for flesh foods it ranged from 0.02 to 15.5 (median 2.2). The SD for eggs ranged from 0.02 to 17.6 (median 3.3). Finally, the SD for vitamin A-rich fruits and vegetables ranged from 2.7 to 11.4 (median 6.7) and for other fruits and vegetables from 0.6 to 12.7 (median 6.2). The distribution of the SDs for each outcome is presented in Figure 3 and Supplementary Figure 11. The district percentages and the between cluster SDs are depicted in the maps in Figure 4 and in Supplementary Figures 12–19.

### Correlation Between District Percent and Cluster Standard Deviation

**Figure 5:**
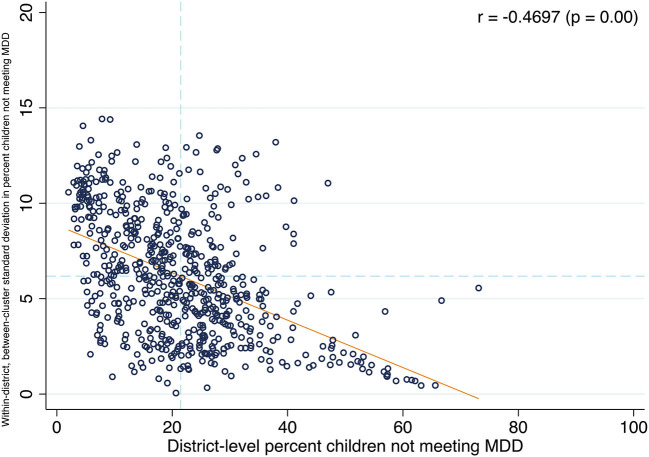
District-level association between percent children not meeting the minimum dietary diversity and within-district, between-cluster standard deviation in percent children not meeting the minimum dietary diversity.

We examined the correlations between the district percentage and cluster SDs for each outcome. We found a positive correlation between the district percentages and cluster SDs for breastfeeding (0.42, *p* < 0.001), and grains, roots, and tubers (0.34, *p* < 0.001). However, we found significant negative correlations for the district percentages and cluster SDs of the overall MDD (-0.47, *p* < 0.001), legumes and nuts (−0.49, *p* < 0.001), flesh foods (–0.52, *p* < 0.001), eggs (–0.59, *p* < 0.001), vitamin A–rich fruits/vegetables (–0.09, *p* = 0.02), and other fruits and vegetables (–0.39, *p* < 0.001). We did not find a significant correlation for the district percent and cluster SD of dairy consumption. These correlation plots are presented in Figure 5 and Supplementary Figures 20–27.

## Discussion

This study had four salient findings. First, we found that clusters were the largest source of geographic variation for children not eating grains, roots, and tubers, legumes and nuts, dairy, vitamin A-rich vegetables and fruits, and other vegetables and fruits. Furthermore, states were the largest source of geographic variation for children not meeting the MDD, not being breastfed, not eating flesh foods, and not eating eggs. Second, we found that the district SDs had wide ranges for all nine outcomes. Third, the districts with the highest percentage of children not meeting the MDD were clustered in Rajasthan and Uttar Pradesh, while districts with the highest percentage of children not eating dairy were clustered in Chhattisgarh, Odisha, and the north east. This underscores the importance of analyzing each component food group as consumption varies geographically. Fourth, we found positive correlations between the district percentages and cluster SDs of a child not being breastfed or not eating grains, roots, and tubers. This implies that for these two food groups, districts with a high percentage of children not consuming these food groups have a larger degree of small area variation. However, we found negative correlations between the district percentages and cluster SDs of children not meeting the MDD, not eating legumes and nuts, not eating flesh foods, not eating eggs, not eating vitamin A-rich fruits/vegetables, and not eating other fruits and vegetables. This implies that districts with a lower percentage of not meeting the MDD, or not eating one of the listed food groups, still have areas within the district that might have much higher percentages of children not meeting the MDD or consuming certain foods, an indication of persistent inequality.

There are four limitations to this study. First, the NFHS-4 asked about the foods consumed in the past 24-h period even though many foods are consumed less frequently than that (36). Second, responses to questions about foods consumed are self-reported, a possible source of measurement error. However, the NFHS are considered to be high quality, which we believe alleviates that concern (37). Third, we acknowledge that dietary diversity measures do not consider the amount of food consumed, which limits our understanding of micronutrient consumption. Fourth, this study uses food groups identified by the World Health Organization. As such, we do not examine consumption of traditional foods that are important sources of vitamin A and protein (38).

These findings are policy relevant for several reasons. First, the relative importance of lower levels has been shown in previous studies in the context of child malnutrition. For example, much of the geographic variation in household poverty and the risk factors for child malnutrition is attributable to cluster and between-cluster variation (19–21). Another study found that most of the geographic variation in child anthropometry and hemoglobin measures was attributable to within-cluster, or household, differences, further underscoring the importance of lower geographic levels (39). Our results further underscore the importance of these lower levels given that the largest share in variation of a child consuming grains, roots, and tubers, legumes and nuts, dairy, vitamin A-rich vegetables and fruits, and other vegetables and fruits is attributable to clusters.

Second, our results showed large SDs for each of the outcomes at the district level. This is meaningful because it indicates that while a district might have a low percentage of children not meeting the MDD, or not eating one of the food groups, there still exist wide variations within the district. This was verified by our correlation analysis, which showed that for most of the outcomes, there was a strong negative correlation between the district percentage and cluster SD of an outcome. For example, while the percentage of children not meeting the MDD in Kanchipuram, a district in the state of Tamil Nadu, was low, the SD in this district was 10.4, which explains why there are clusters within Kanchipuram where the percentage of children not meeting the MDD is higher. In one cluster the percentage was 62.2% while in another it was 72.5%. That the correlations between the district percentages and cluster SDs for so many of the outcomes were negative could be due the fact that the drivers of dietary diversity are complex (33), and that household income, a key determinant of dietary diversity (40–42), varies between villages within states (21). These results further emphasize the importance of looking within districts given that the NNS typically selects poor-performing districts. In addition to targeting low-performing districts, those areas within high-performing districts that perform poorly should also be prioritized. Therefore, future research should examine the district and cluster level factors that might explain this small area variation in dietary diversity. Doing so could help lower within district inequality and improve overall nutrition outcomes, especially considering that India's Integrated Child Development Services (ICDS) already implements interventions at the village level (19). Our results can help state-level policy makers identify the specific determinants of dietary diversity within district and between villages. These determinants, such as female literacy, agricultural production, and household socioeconomic status can then be addressed through the already functioning ICDS platform.

Third, in addition to targeting lower geographic levels, our results highlight the importance of examining each of the component food groups that makes up the MDD. Doing so is important given that the percentage of children not eating a given food varies geographically. For example, the districts with the highest percentage of children not breastfeeding were clustered in the south (Karnataka and Tamil Nadu), while the districts with the highest percentage of children of not eating other fruits and vegetables were clustered in the west and north (Rajasthan, Uttar Pradesh, and Bihar). Furthermore, the percentage of children not eating flesh foods, eggs, and dairy were high across all 640 districts, each of which are food groups associated with improved child growth outcomes (28, 43–47).

## Data Availability Statement

Publicly available datasets were analyzed in this study. This data can be found here: the Indian Demographic Health Survey can be downloaded from: https://dhsprogram.com/.

## Ethics Statement

Ethical review and approval was not required for the study on human participants in accordance with the local legislation and institutional requirements. Written informed consent to participate in this study was provided by the participants' legal guardian/next of kin.

## Author Contributions

AJ, RK, and SS: conceptualization, design, and data interpretation. AJ and WW: data acquisition and analysis. AJ: drafting of the manuscript. AJ, WW, KJ, RS, RK, and SS: critical revisions to manuscript. RK and SS: overall supervision. All authors contributed to the article and approved the submitted version.

## Funding

This research was funded by Bill and Melinda Gates Foundation, INV-002992.

## Conflict of Interest

The authors declare that the research was conducted in the absence of any commercial or financial relationships that could be construed as a potential conflict of interest.

## Publisher's Note

All claims expressed in this article are solely those of the authors and do not necessarily represent those of their affiliated organizations, or those of the publisher, the editors and the reviewers. Any product that may be evaluated in this article, or claim that may be made by its manufacturer, is not guaranteed or endorsed by the publisher.
