# Supplementary material for: Small Area Variations in Dietary Diversity Among Children in India: A Multilevel Analysis of 6–23-Month-Old Children
Source: Front Nutr. 2022 Feb 16;8:791509. doi: 10.3389/fnut.2021.791509 (PMC8890590; doi:10.3389/fnut.2021.791509)
Supplement: Supplementary file 1 [file Data_Sheet_1.pdf]

Small Area Variations in Dietary Diversity Among Children in India: A Multilevel Analysis of 6-23-Month-Old Children

Supplementary Materials

Citation: *Jain A, Wang W, James KS, Sarwal R, Kim R and Subramanian SV. (2022). Small Area Variations in Dietary Diversity Among Children in India: A Multilevel Analysis of 6–23-Month-Old Children. Front. Nutr. 8:791509. doi: 10.3389/fnut.2021.791509*

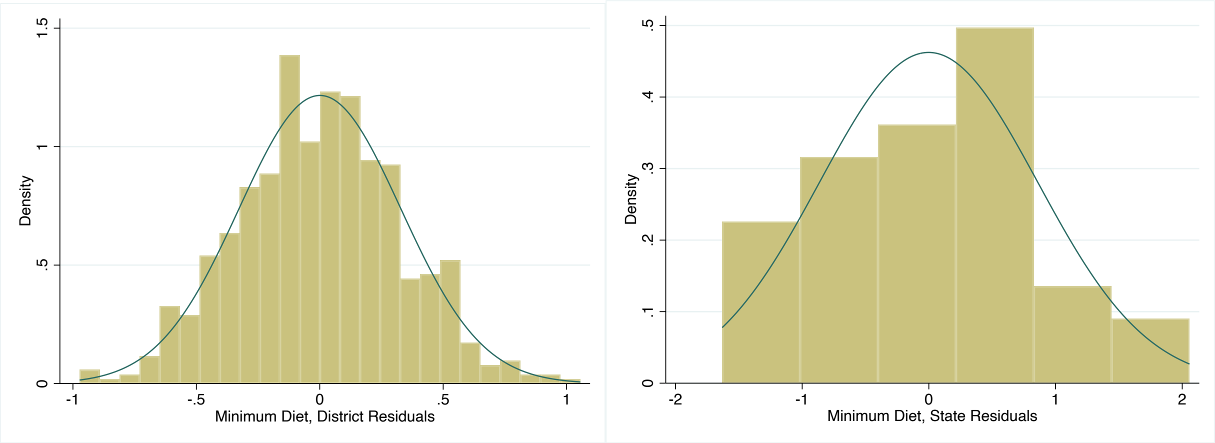

Supplementary Figure 1: Distribution of district and state residuals for not meeting the MDD

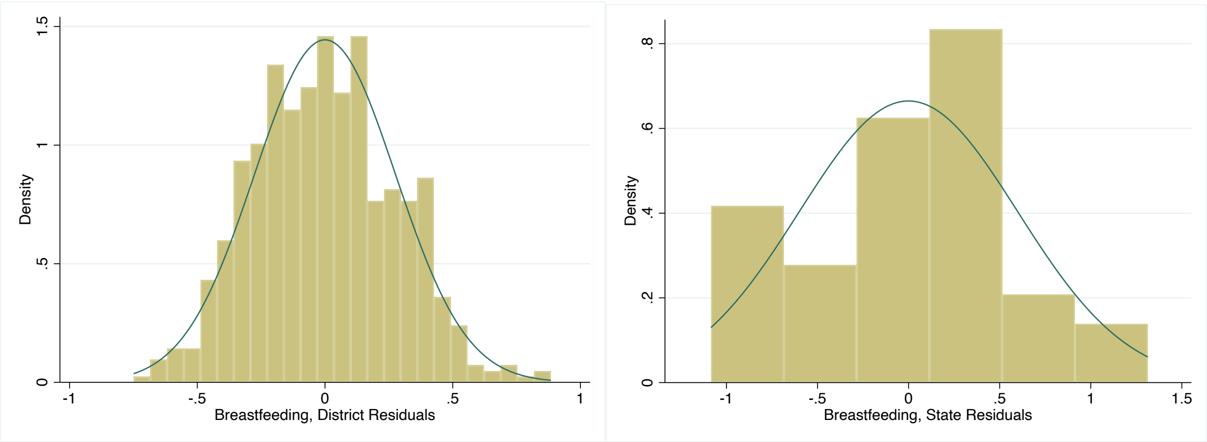

Supplementary Figure 2: Distribution of district and state residuals for not breastfeeding

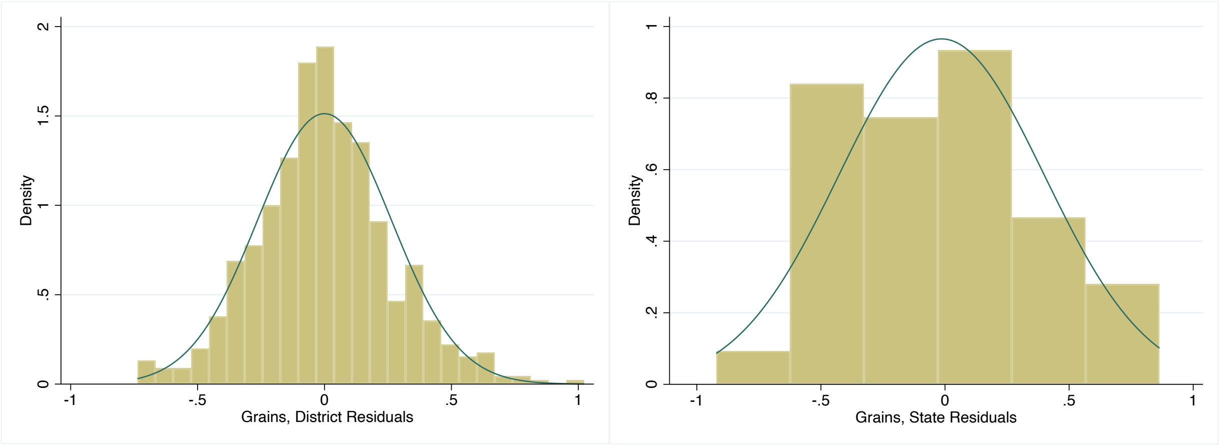

Supplementary Figure 3: Distribution of district and state residuals for not eating grains

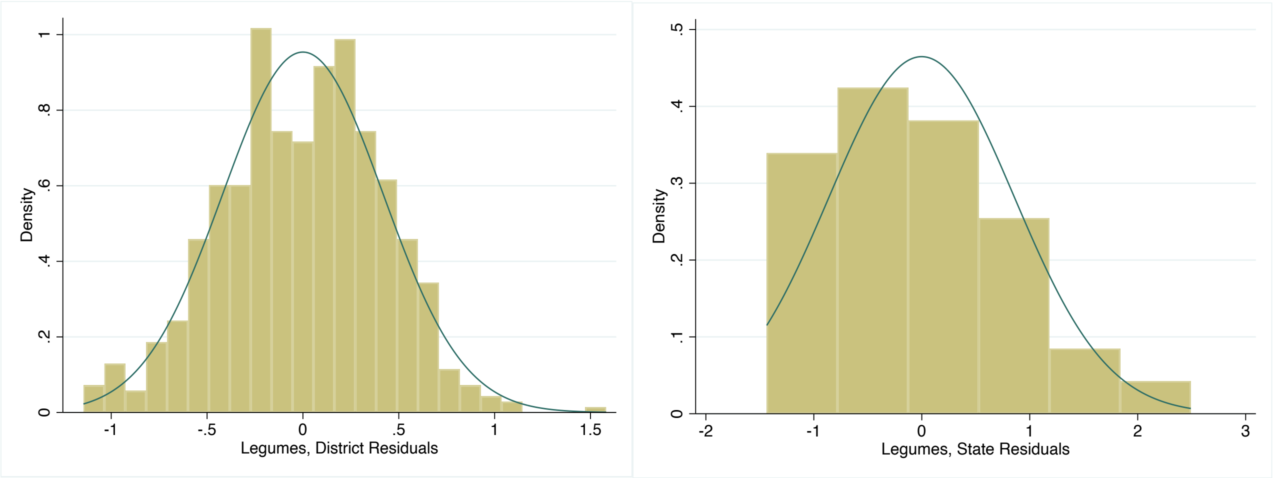

Supplementary Figure 4: Distribution of district and state residuals for not eating legumes

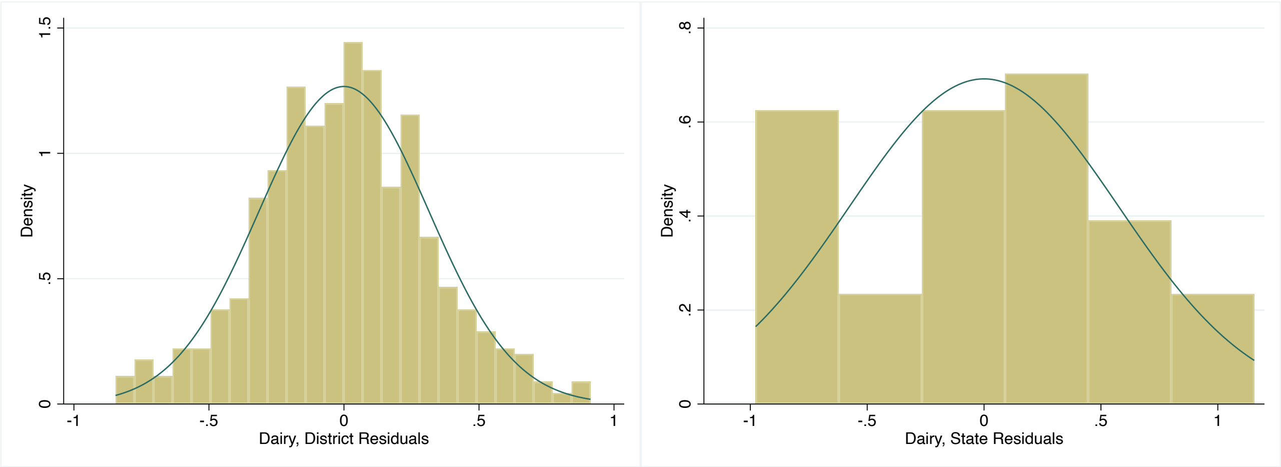

Supplementary Figure 5: Distribution of district and state residuals for not consuming dairy

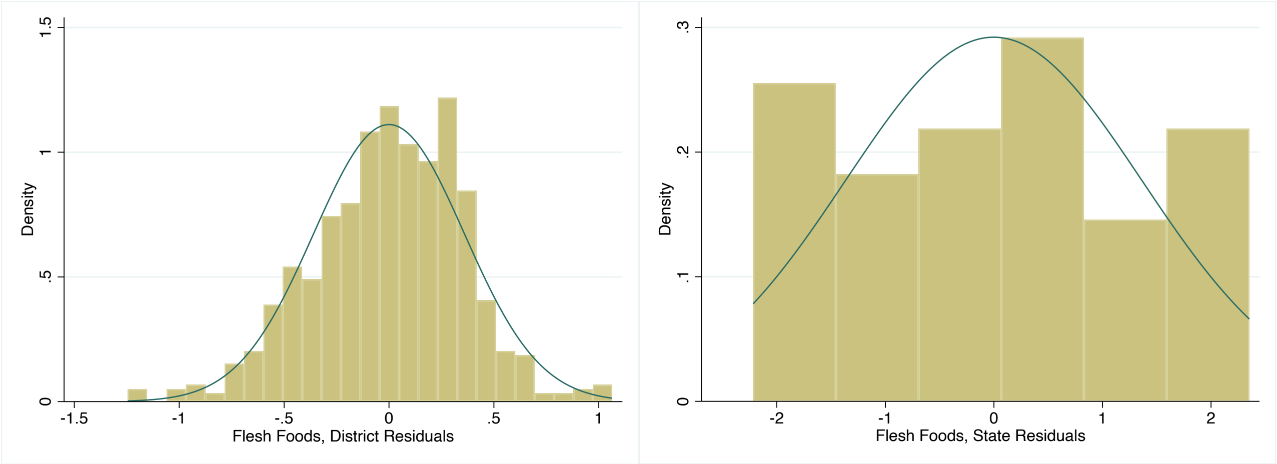

Supplementary Figure 6: Distribution of district and state residuals for not eating flesh foods

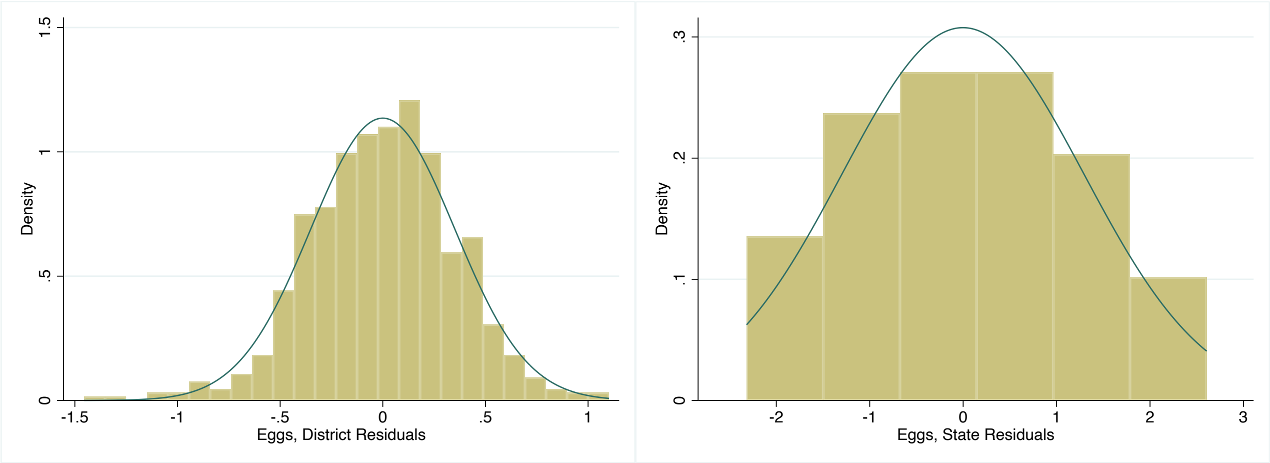

Supplementary Figure 7: Distribution of district and state residuals for not eating eggs

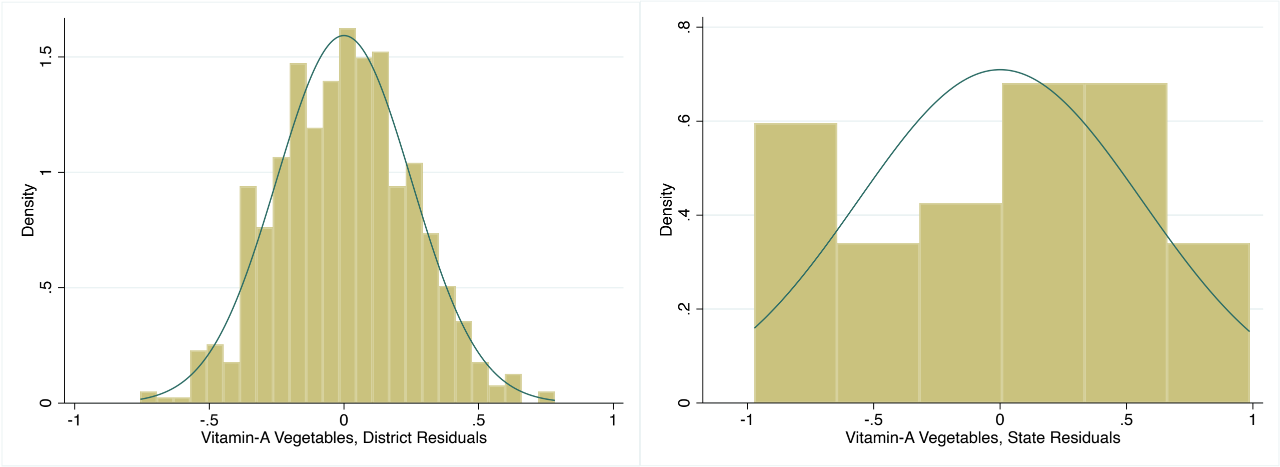

Supplementary Figure 8: Distribution of district and state residuals for not eating vitamin-A vegetables

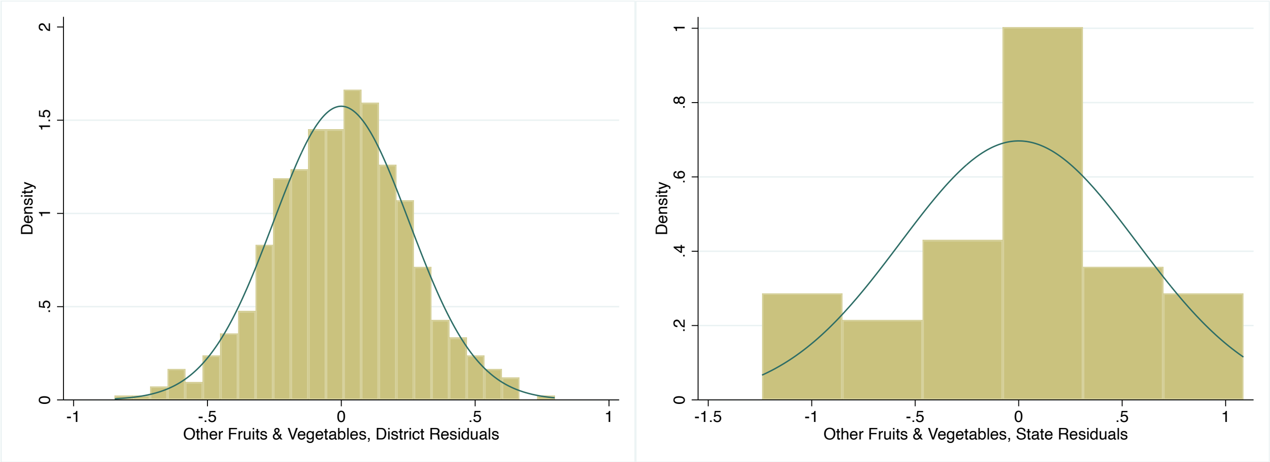

Supplementary Figure 9: Distribution of district and state residuals for not eating other fruits & vegetables

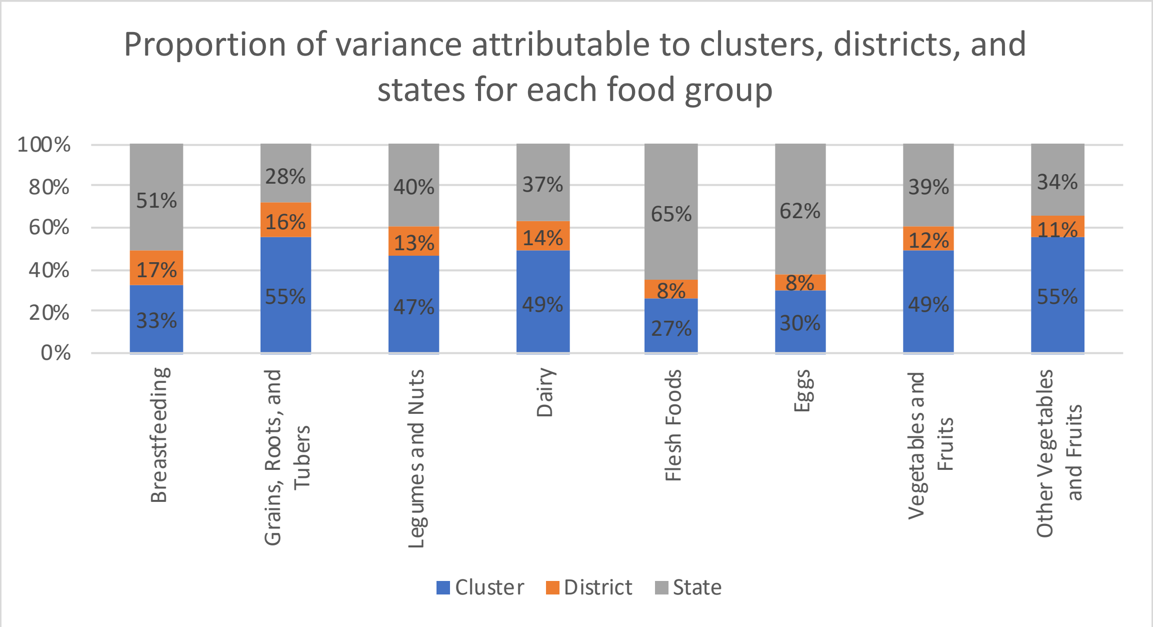

Supplementary figure 10: Variance partitioned between clusters, districts, and states for not consuming each food group

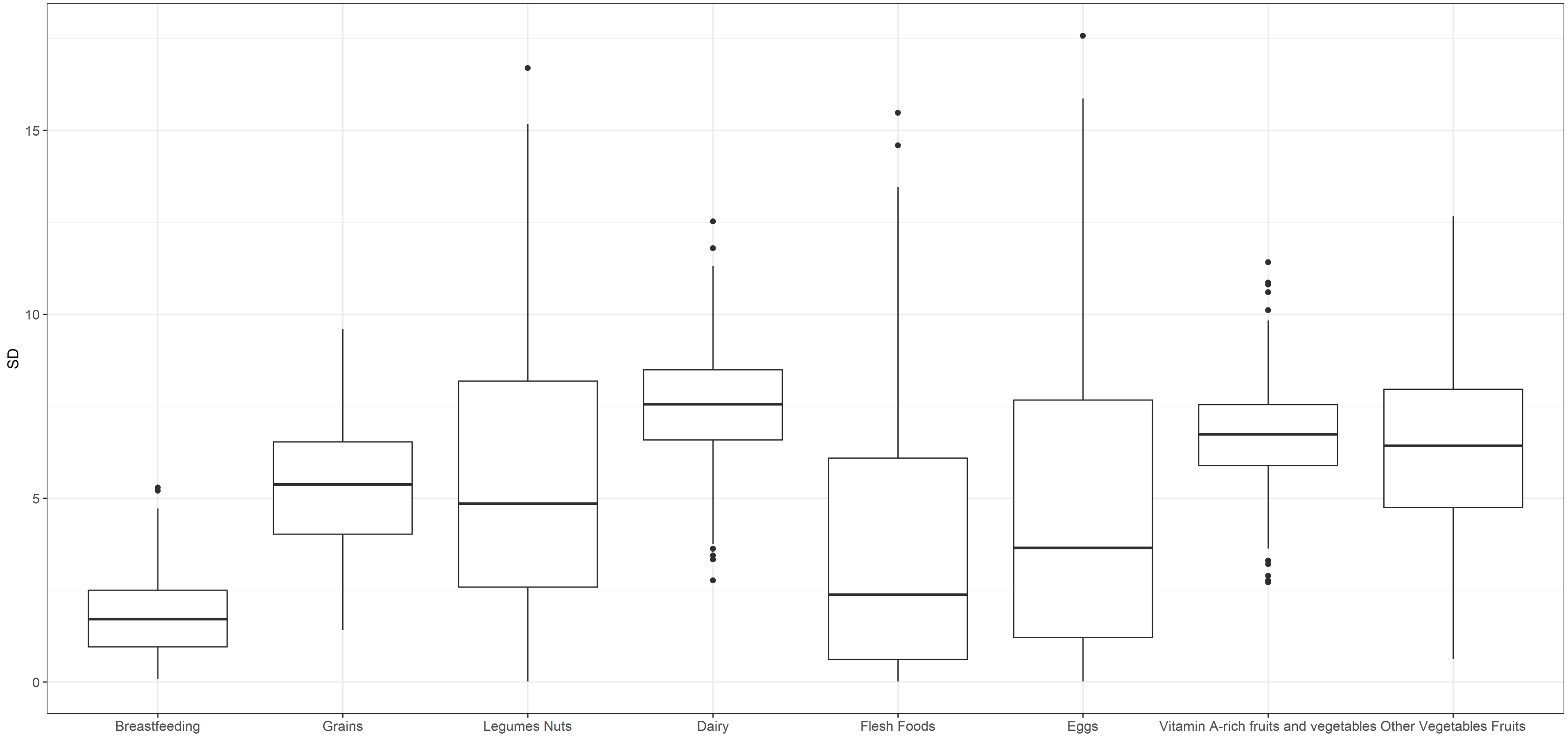

Supplementary figure 11: Distribution of standard deviations for not consuming each food group

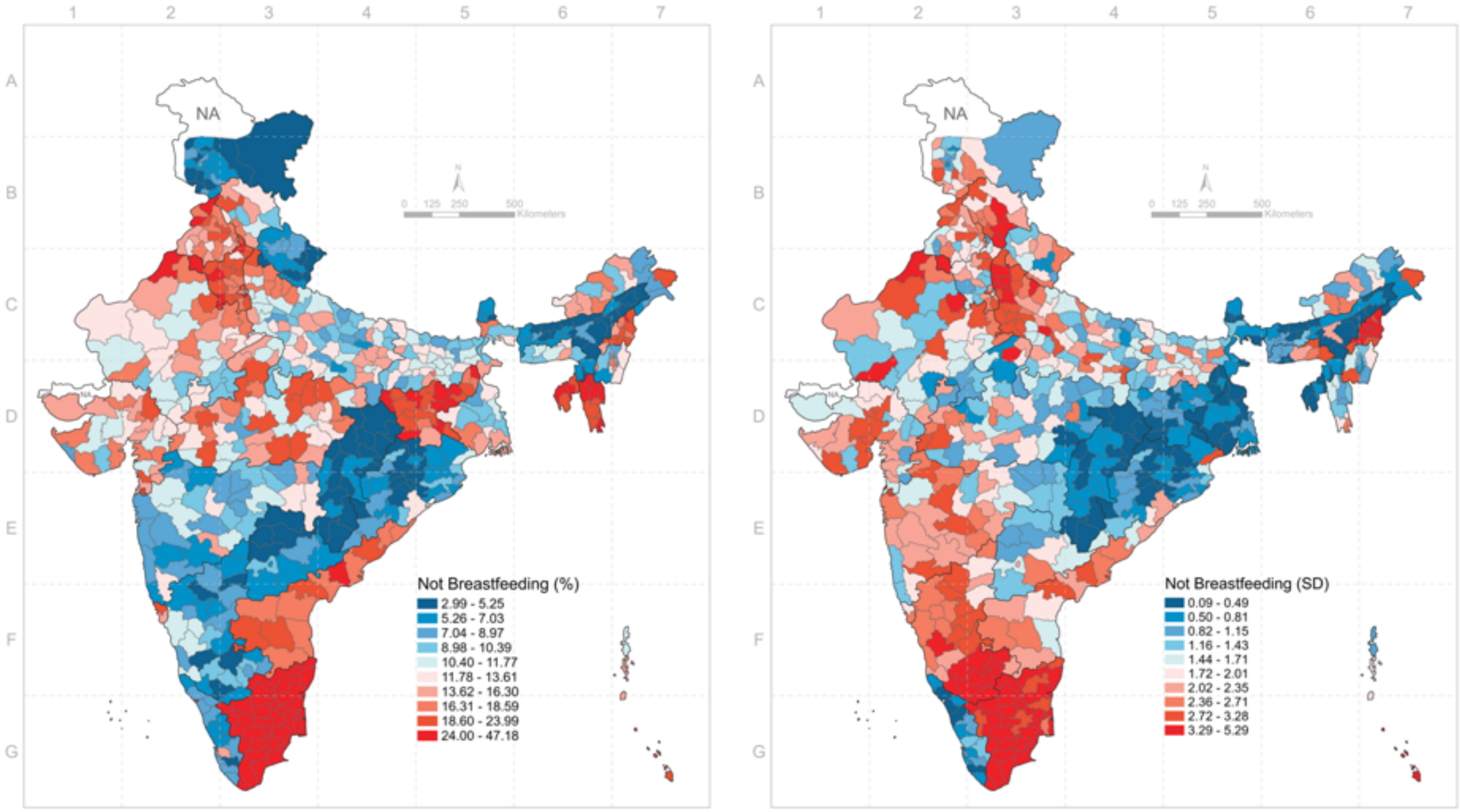

Supplementary Figure 12: (A) Geographic distribution of percent children not breastfeeding across 640 districts in India | (B) Geographic distribution of within-district, between-cluster standard deviation in percent children not breastfeeding across 640 districts in India

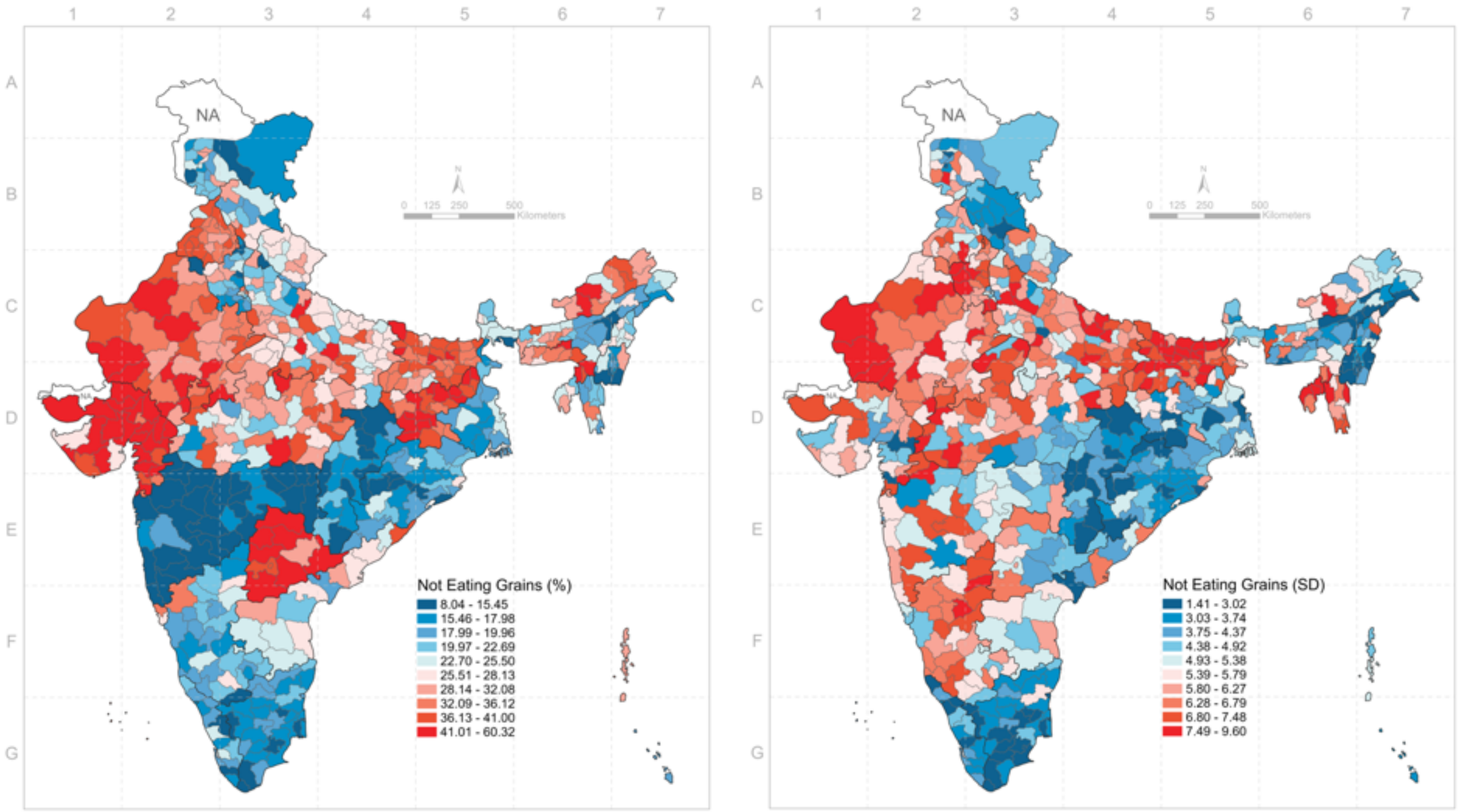

Supplementary figure 13: (A) Geographic distribution of percent children not eating grains across 640 districts in India | (B) Geographic distribution of within-district, between-cluster standard deviation in percent children not eating grains across 640 districts in India

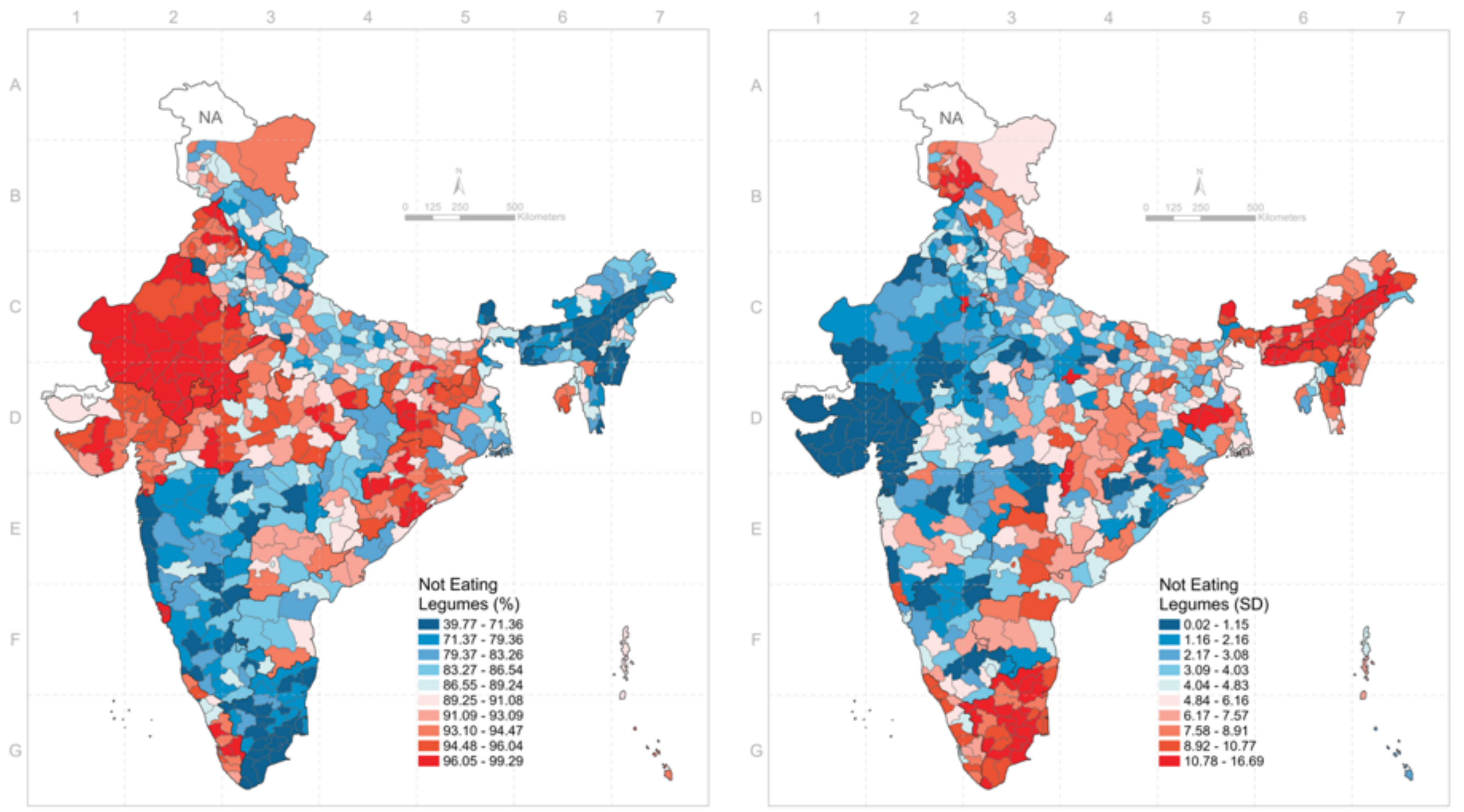

Supplementary figure 14: (A) Geographic distribution of percent children not eating legumes across 640 districts in India | (B) Geographic distribution of within-district, between-cluster standard deviation in percent children not eating legumes across 640 districts in India

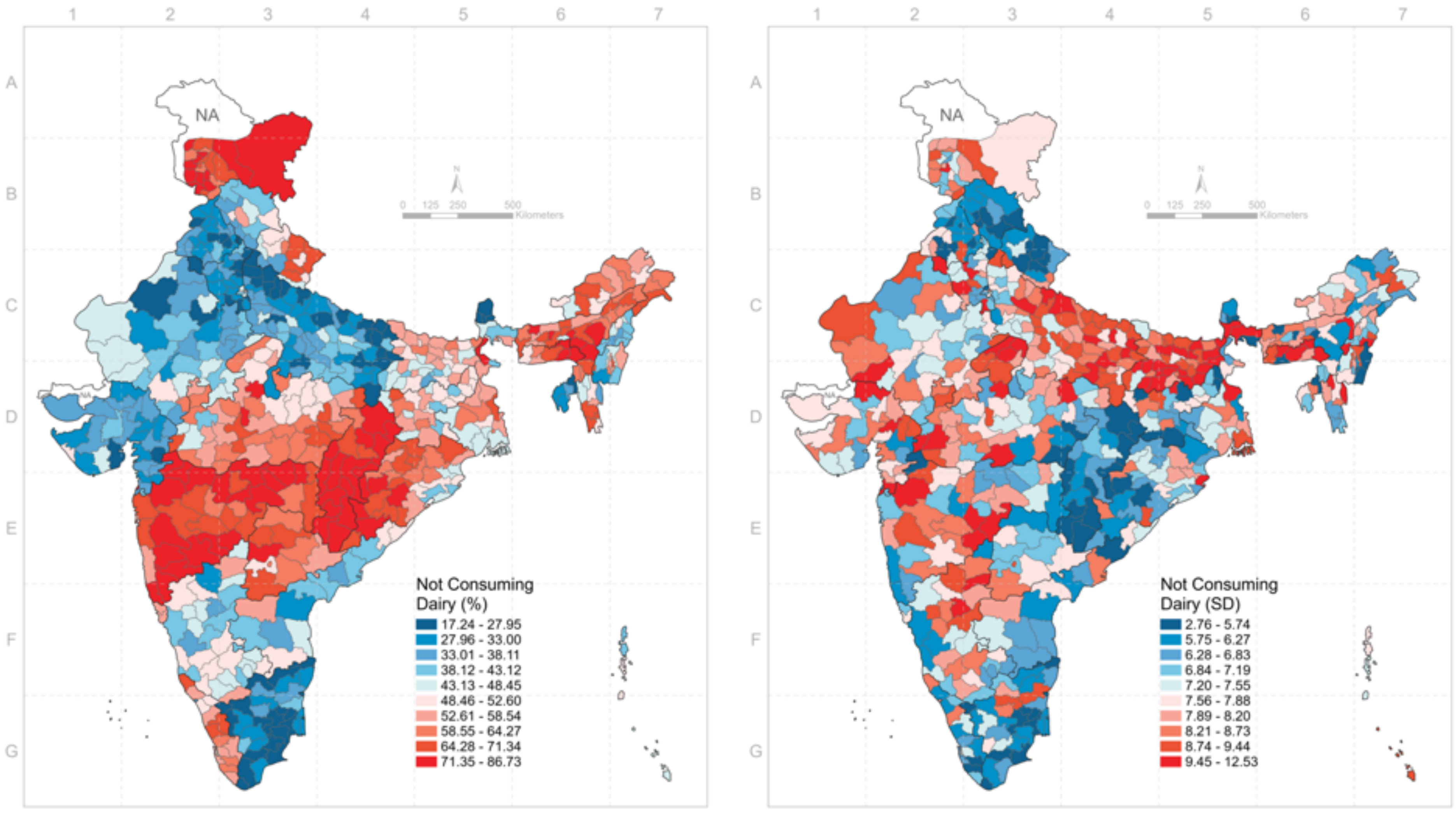

Supplementary figure 15: (A) Geographic distribution of percent children not consuming dairy across 640 districts in India | (B) Geographic distribution of within-district, between-cluster standard deviation in percent children not consuming dairy across 640 districts in India

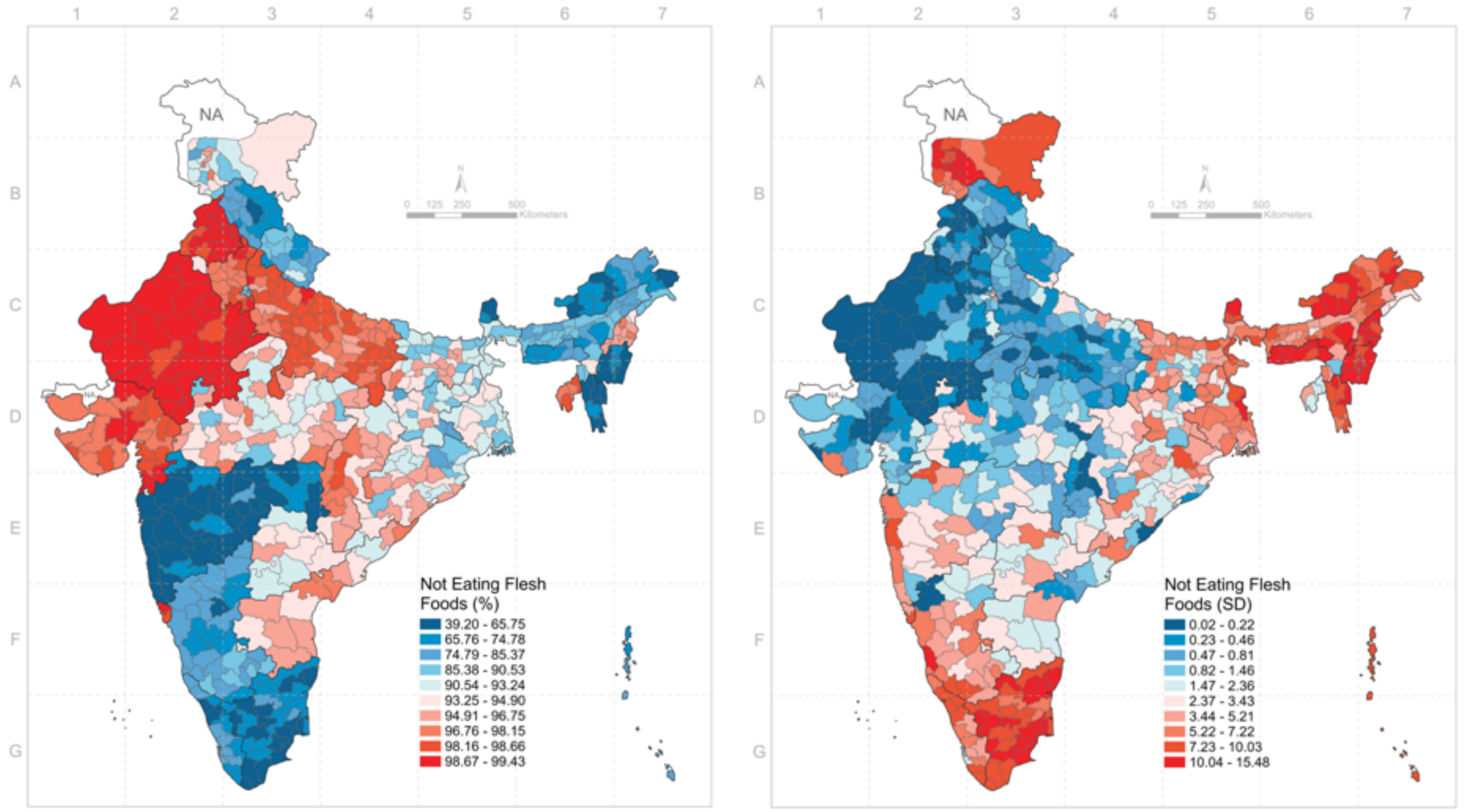

Supplementary figure 16: (A) Geographic distribution of percent children not eating flesh foods across 640 districts in India | (B) Geographic distribution of within-district, between-cluster standard deviation in percent children not eating flesh foods across 640 districts in India

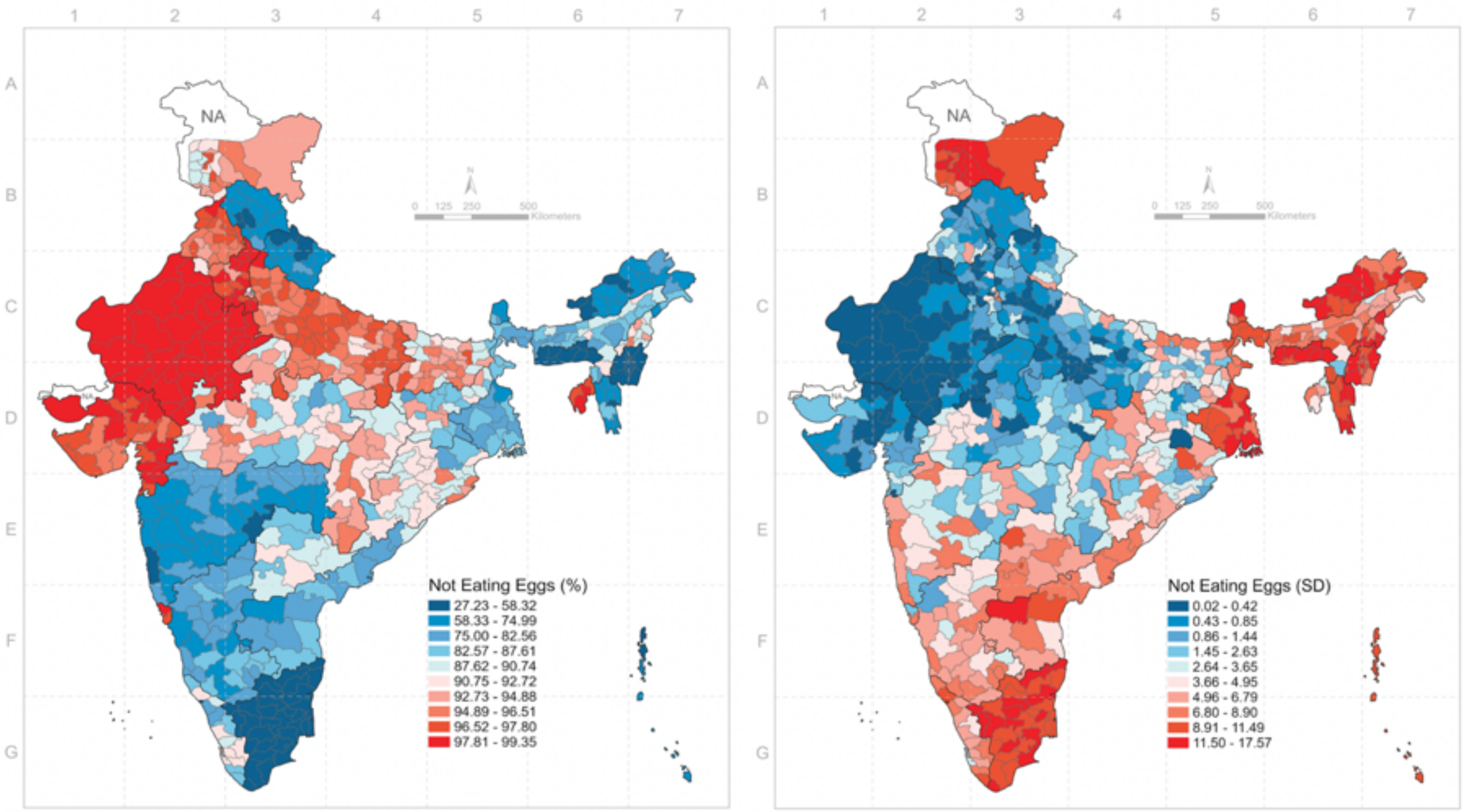

Supplementary figure 17: (A) Geographic distribution of percent children not eating eggs across 640 districts in India | (B) Geographic distribution of within-district, between-cluster standard deviation in percent children not eating eggs across 640 districts in India

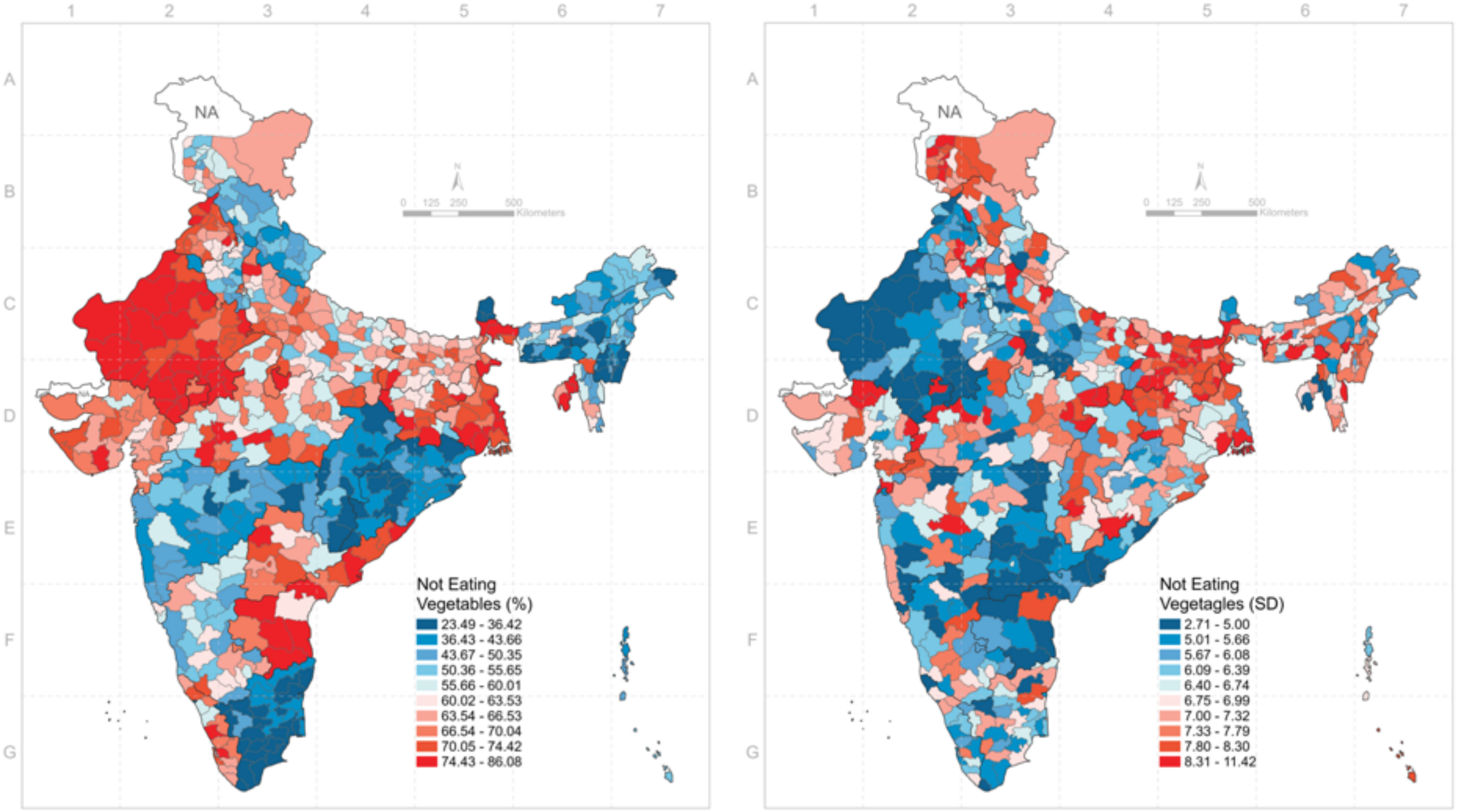

Supplementary figure 18: (A) Geographic distribution of percent children not eating vitamin-A rich vegetables across 640 districts in India | (B) Geographic distribution of within-district, between-cluster standard deviation in percent children not eating vitamin-A rich vegetables across 640 districts in India

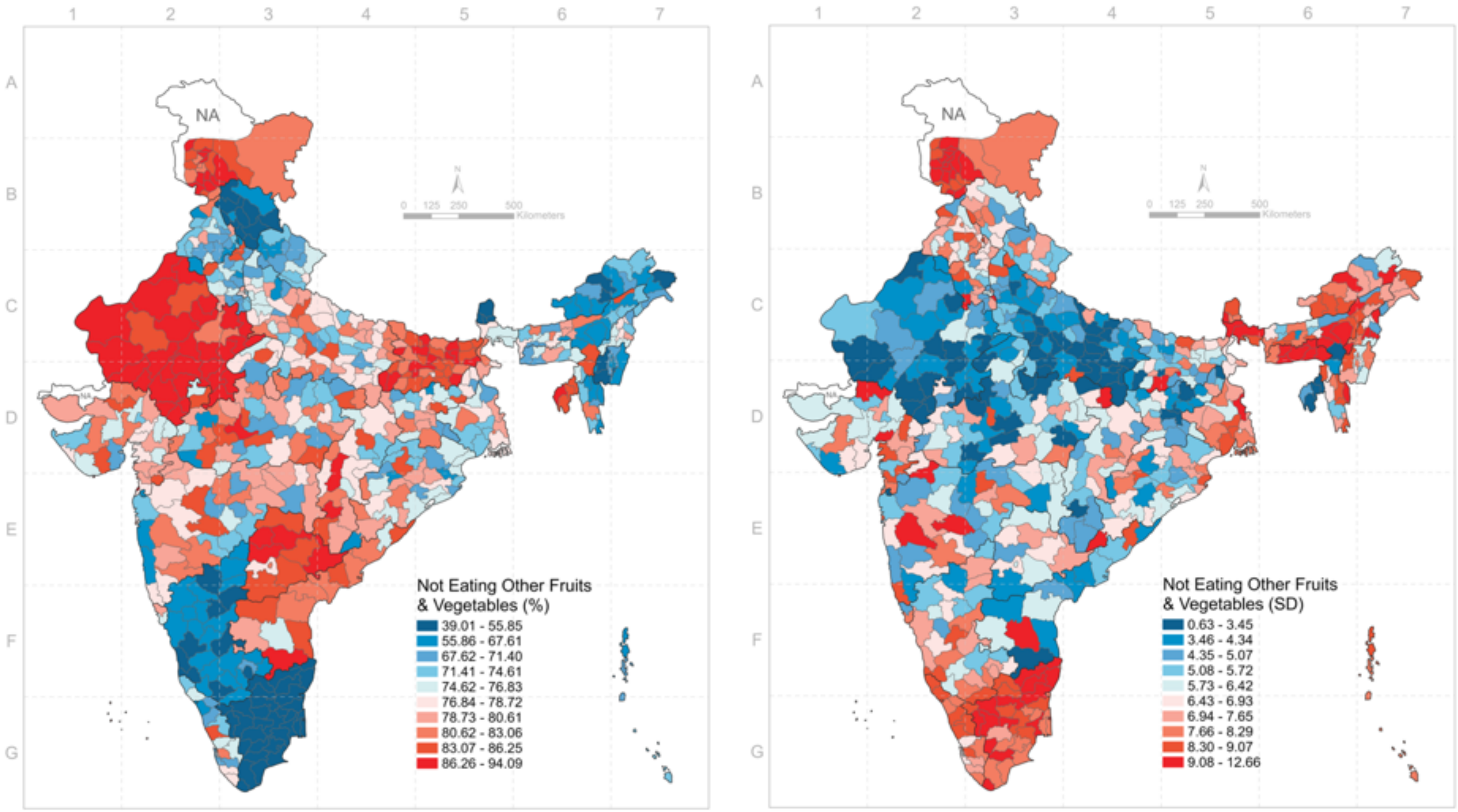

Supplementary figure 19: (A) Geographic distribution of percent children not eating other fruits & vegetables across 640 districts in India | (B) Geographic distribution of within-district, between-cluster standard deviation in percent children not eating other fruits & vegetables across 640 districts in India

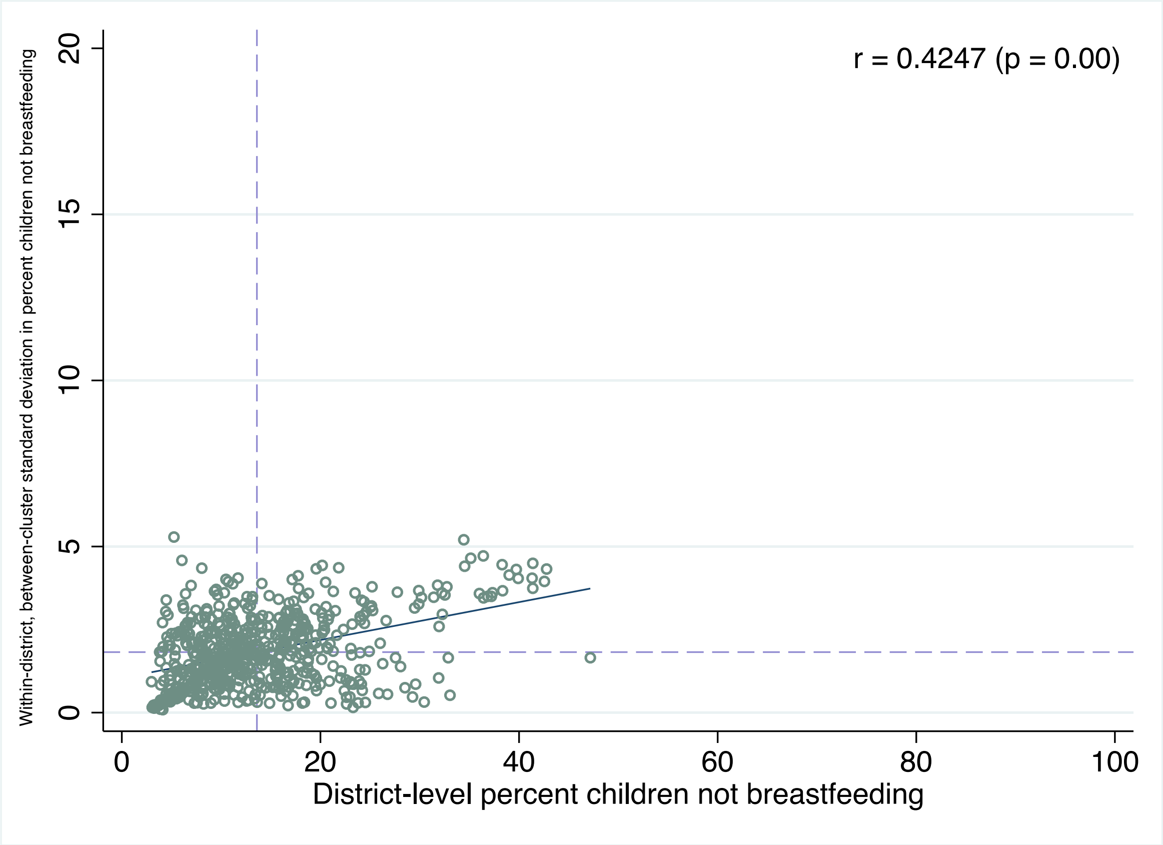

Supplementary figure 20: District-level association between percent children not breastfeeding and within-district, between-cluster standard deviation in percent children not breastfeeding

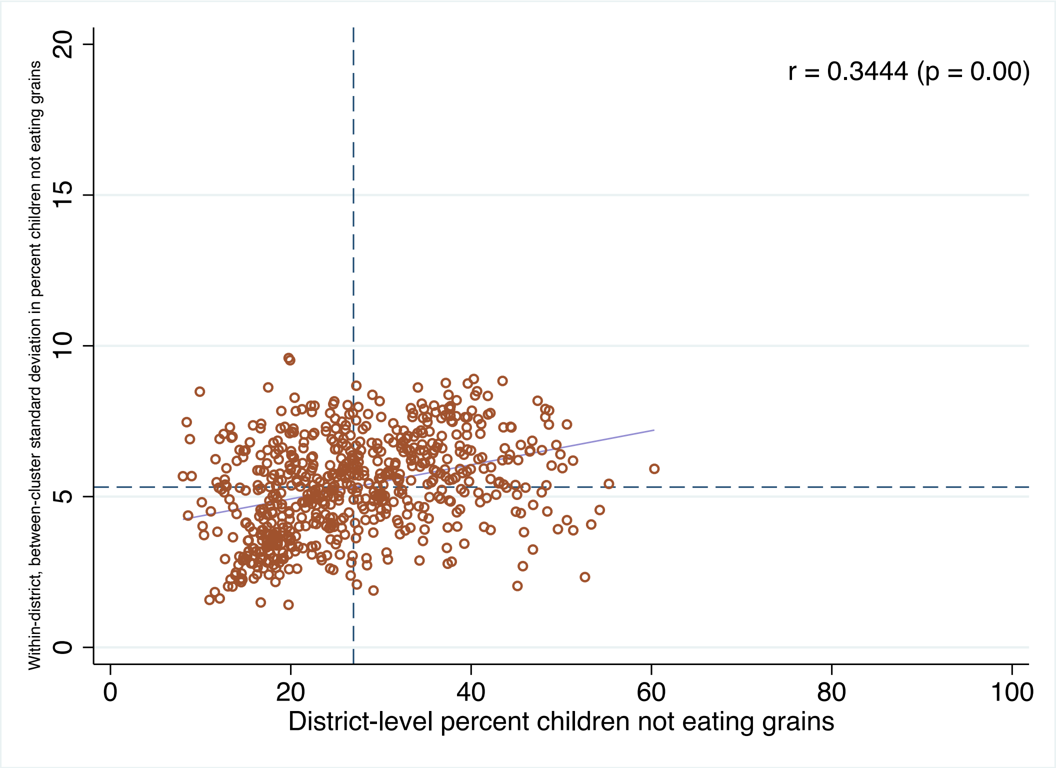

Supplementary figure 21: District-level association between percent children not eating grains and within-district, between-cluster standard deviation in percent children not eating grains

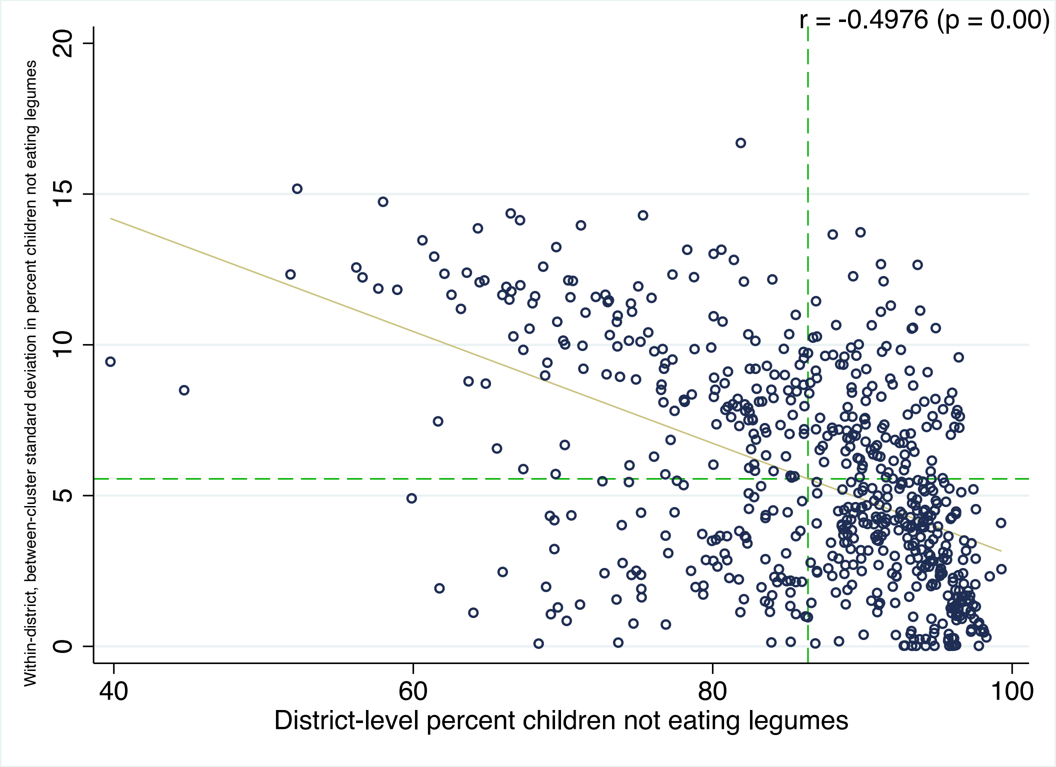

Supplementary figure 22: District-level association between percent children not eating legumes and within-district, between-cluster standard deviation in percent children not eating legumes

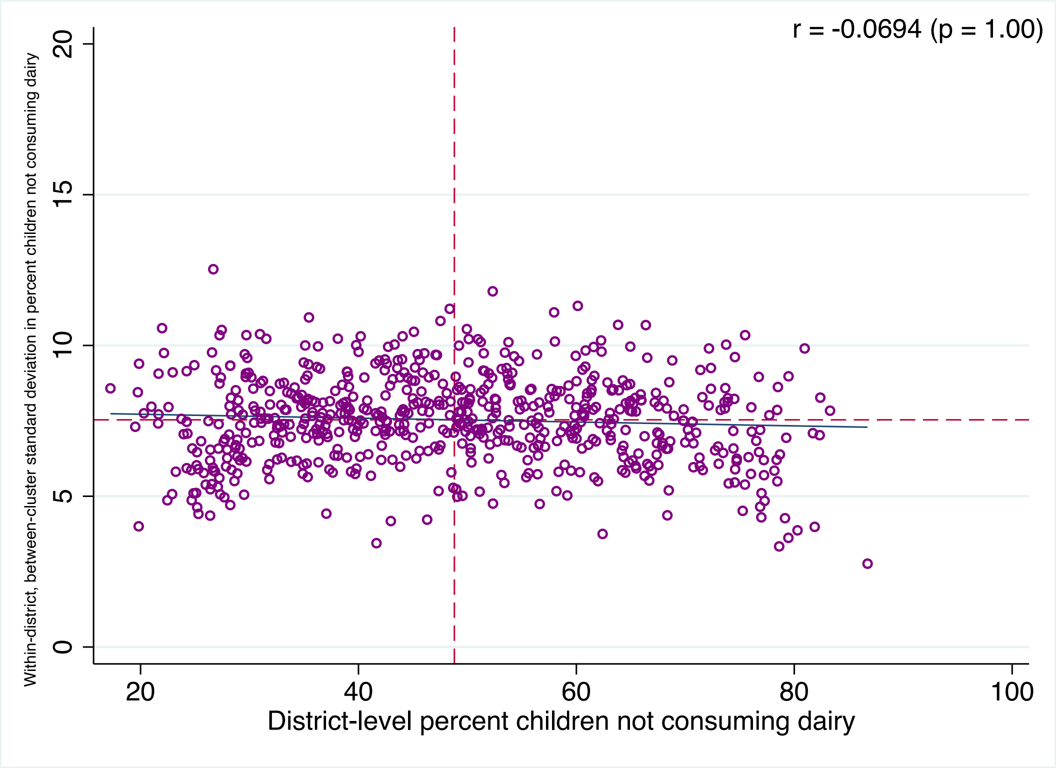

Supplementary figure 23: District-level association between percent children not consuming dairy and within-district, between-cluster standard deviation in percent children not consuming dairy

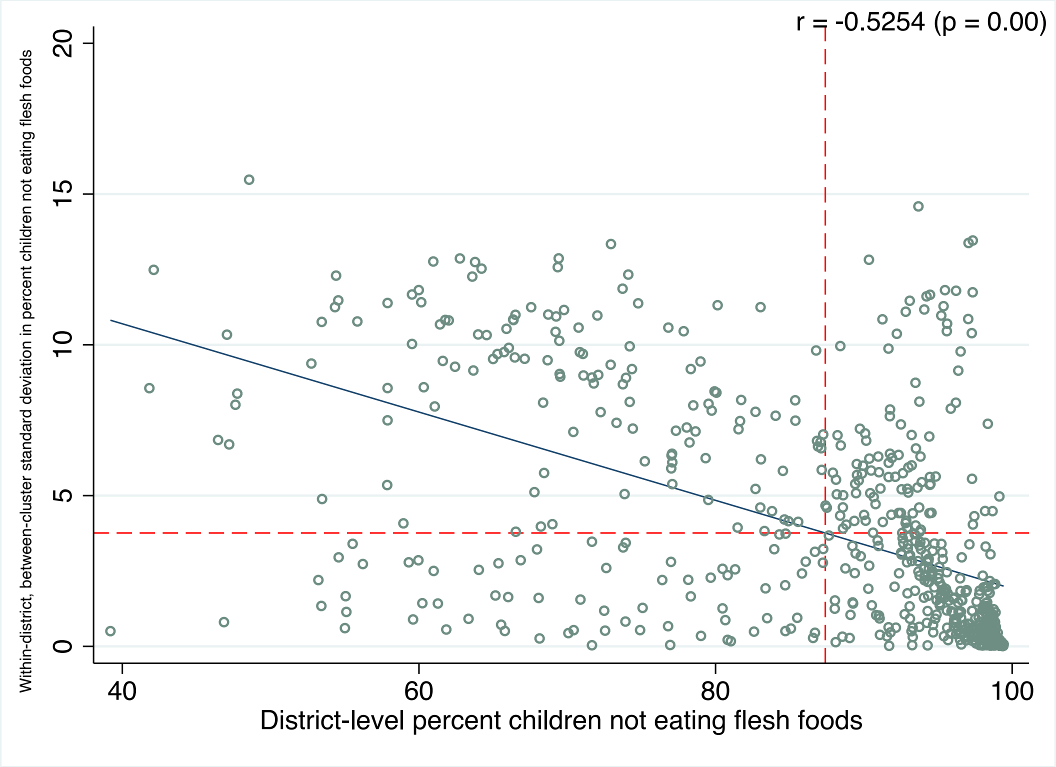

Supplementary figure 24: District-level association between percent children not eating flesh foods and within-district, between-cluster standard deviation in percent children not eating flesh foods

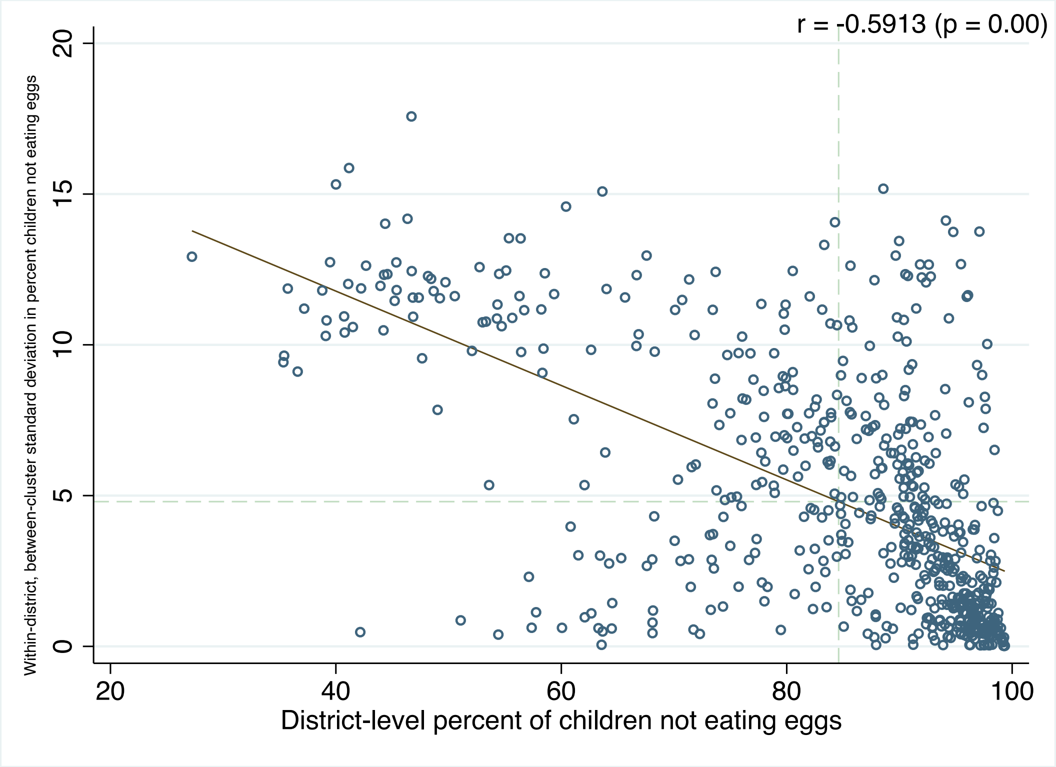

Supplementary figure 25: District-level association between percent children not eating eggs and within-district, between-cluster standard deviation in percent children not eating eggs

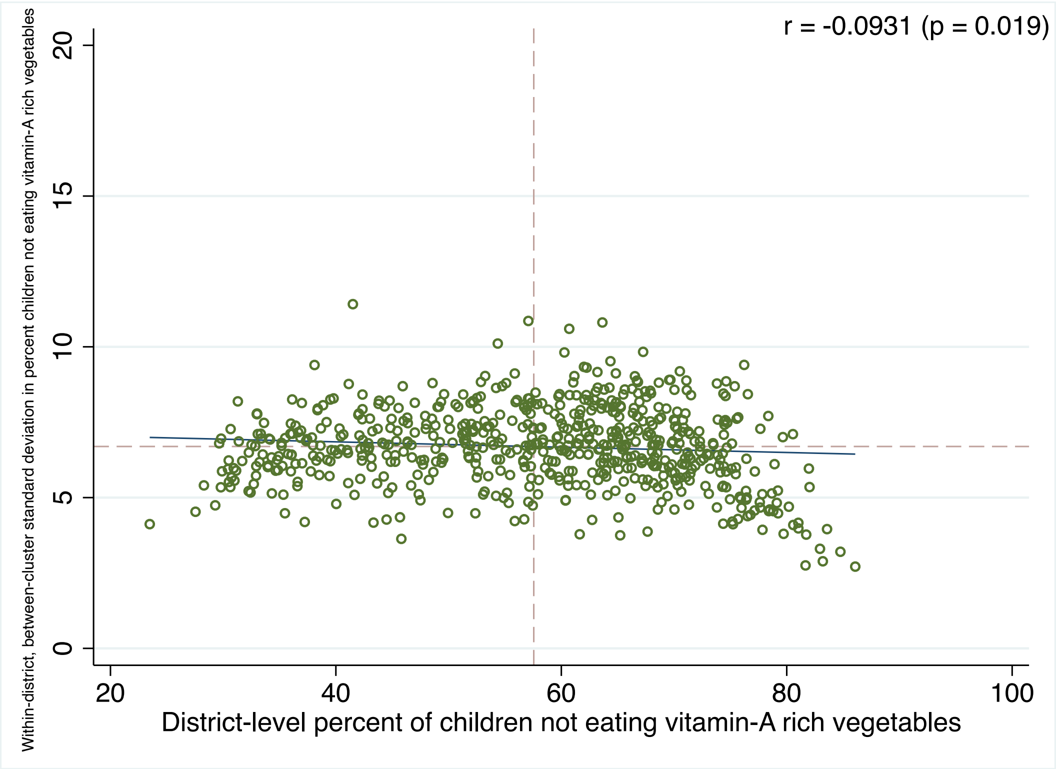

Supplementary figure 26: District-level association between percent children not eating vitamin-A rich vegetables and within-district, between-cluster standard deviation in percent children not eating vitamin-A rich vegetables

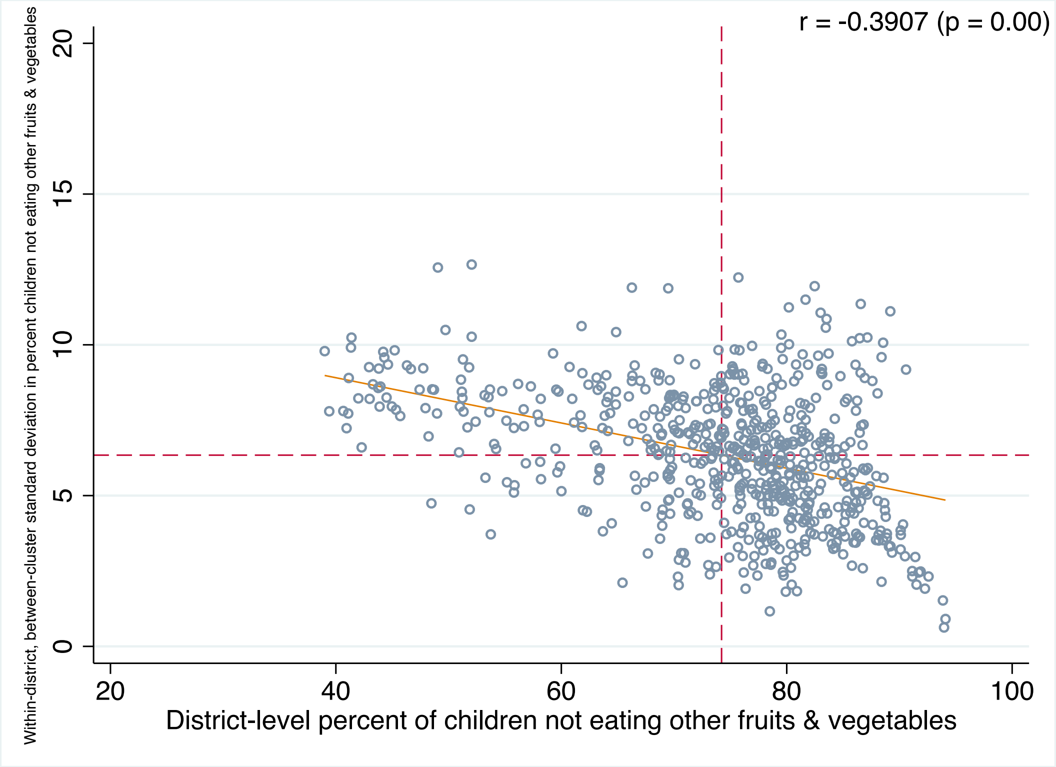

Supplementary figure 27: District-level association between percent children not eating other fruits & vegetables and within-district, between-cluster standard deviation in percent children not eating other fruits & vegetables
